# Soluble receptor for advanced glycation end products (sRAGE) as a biomarker of COPD

**DOI:** 10.1186/s12931-021-01686-z

**Published:** 2021-04-27

**Authors:** Katherine A. Pratte, Jeffrey L. Curtis, Katerina Kechris, David Couper, Michael H. Cho, Edwin K. Silverman, Dawn L. DeMeo, Frank C. Sciurba, Yingze Zhang, Victor E. Ortega, Wanda K. O’Neal, Lucas A. Gillenwater, David A. Lynch, Eric A. Hoffman, John D. Newell, Alejandro P. Comellas, Peter J. Castaldi, Bruce E. Miller, Simon D. Pouwels, Nick H. T. ten Hacken, Rainer Bischoff, Frank Klont, Prescott G. Woodruff, Robert Paine, R. Graham Barr, John Hoidal, Claire M. Doerschuk, Jean-Paul Charbonnier, Ruby Sung, Nicholas Locantore, John G. Yonchuk, Sean Jacobson, Ruth Tal-singer, Debbie Merrill, Russell P. Bowler

**Affiliations:** 1 Department of Biostatistics, National Jewish Health, Denver, CO USA; 2 Department of Internal Medicine, University of Michigan Health System, Ann Arbor, MI USA; 3 Medical Service, Ann Arbor Healthcare System, Ann Arbor, MI USA; 4 Department of Biostatistics and Informatics, School of Public Health, University of Colorado Denver, Anschutz Medical Campus, Aurora, CO USA; 5 Department of Biostatistics, Collaborative Studies Coordinating Center, University of North Carolina at Chapel Hill, Chapel Hill, NC USA; 6 Channing Division of Network Medicine, Brigham and Women’s Hospital, Boston, MA USA; 7 Division of Pulmonary and Critical Care Medicine, Brigham and Women’s Hospital, Boston, MA USA; 8 Department of Medicine, University of Pittsburgh, Pittsburgh, PA USA; 9 Center for Genomics and Personalized Medicine Research, Wake Forest School of Medicine, Winston-Salem, NC USA; 10 Marsico Lung Institute (CF Research Center), University of North Carolina at Chapel Hill, Chapel Hill, NC USA; 11 Division of Pulmonary Medicine, Department of Medicine, National Jewish Health, 1400 Jackson Street, Denver, CO 80206 USA; 12 Computational Bioscience Program, University of Colorado Anschutz Medical Campus, Aurora, CO 80045 USA; 13 Department of Radiology, National Jewish Health, Denver, CO USA; 14 Department of Radiology and Biomedical Engineering, University of Iowa, Iowa City, IA USA; 15 Department of Internal Medicine, College of Medicine, University of Iowa Carver, Iowa City, IA USA; 16 Research and Development, GlaxoSmithKline, Collegeville, PA USA; 17 Department of Pathology and Medical Biology, University of Groningen, Groningen, Netherlands; 18 Department of Analytical Biochemistry, University of Groningen, Groningen, Netherlands; 19 Division of Pulmonary, Critical Care, Sleep and Allergy, Department of Medicine, University of California-San Francisco, San Francisco, CA USA; 20 Cardiovascular Research Institute, University of California-San Francisco, San Francisco, CA USA; 21 Division of Pulmonary and Critical Care, University of Utah, Salt Lake City, UT USA; 22 Division of Pulmonary, Allergy, and Critical Care Medicine, Department of Medicine, Columbia University, New York, NY USA; 23 Thirona, LungQ, Nijmegen, Netherlands; 24 Department of Genetics, National Jewish Health, Denver, CO USA; 25 COPD Foundation, Miami, FL USA

**Keywords:** COPD, Emphysema, Biomarkers, Progression

## Abstract

**Background:**

Soluble receptor for advanced glycation end products (sRAGE) is a proposed emphysema and airflow obstruction biomarker; however, previous publications have shown inconsistent associations and only one study has investigate the association between sRAGE and emphysema. No cohorts have examined the association between sRAGE and progressive decline of lung function. There have also been no evaluation of assay compatibility, receiver operating characteristics, and little examination of the effect of genetic variability in non-white population. This manuscript addresses these deficiencies and introduces novel data from Pittsburgh COPD SCCOR and as well as novel work on airflow obstruction. A meta-analysis is used to quantify sRAGE associations with clinical phenotypes.

**Methods:**

sRAGE was measured in four independent longitudinal cohorts on different analytic assays: COPDGene (n = 1443); SPIROMICS (n = 1623); ECLIPSE (n = 2349); Pittsburgh COPD SCCOR (n = 399). We constructed adjusted linear mixed models to determine associations of sRAGE with baseline and follow up forced expiratory volume at one second (FEV_1_) and emphysema by quantitative high-resolution CT lung density at the 15th percentile (adjusted for total lung capacity).

**Results:**

Lower plasma or serum sRAGE values were associated with a COPD diagnosis (P < 0.001), reduced FEV_1_ (P < 0.001), and emphysema severity (P < 0.001). In an inverse-variance weighted meta-analysis, one SD lower log_10_-transformed sRAGE was associated with 105 ± 22 mL lower FEV_1_ and 4.14 ± 0.55 g/L lower adjusted lung density. After adjusting for covariates, lower sRAGE at baseline was associated with greater FEV_1_ decline and emphysema progression only in the ECLIPSE cohort. Non-Hispanic white subjects carrying the rs2070600 minor allele (A) and non-Hispanic African Americans carrying the rs2071288 minor allele (A) had lower sRAGE measurements compare to those with the major allele, but their emphysema-sRAGE regression slopes were similar.

**Conclusions:**

Lower blood sRAGE is associated with more severe airflow obstruction and emphysema, but associations with progression are inconsistent in the cohorts analyzed. In these cohorts, genotype influenced sRAGE measurements and strengthened variance modelling. Thus, genotype should be included in sRAGE evaluations.

**Supplementary Information:**

The online version contains supplementary material available at 10.1186/s12931-021-01686-z.

## Clinical relevance

The soluble receptor for advanced glycation end products (sRAGE) is an emerging blood protein biomarker for emphysema and chronic obstructive pulmonary disease (COPD).

This study uses four independent cohorts and four distinct sRAGE assay platforms to confirm that sRAGE is an independent blood biomarker for the presence and severity of both emphysema and COPD; however, the association between baseline sRAGE and emphysema progression or COPD progression is less consistent. Furthermore, although there is correlation among different sRAGE assay platforms, many platforms, but not all, have variable sRAGE detection dependent on a subject’s genotype, suggesting that the genetic background should be considered when interpreting sRAGE measurements.

## Background

The Receptor for Advanced Glycation End Products (RAGE; UniProtKB—Q15109) is a 41-kD multi-ligand transmembrane receptor belonging to the immunoglobulin gene superfamily [1]. Cleavage of the extracellular domain of RAGE results in a 35 kD soluble RAGE (sRAGE), which can be measured in plasma or serum. Little is known about the exact functions of sRAGE; however, lower levels of sRAGE have been reported to be associated with increase risk of chronic diseases such as diabetes [2], atherosclerosis [3], coronary artery disease [2], diabetic retinopathy [4], and chronic obstructive pulmonary disease (COPD) [5]. Elevated sRAGE indicated alveolar epithelial cell injury in infection-related ARDS [6] and in diabetic nephropathy [7, 8]. sRAGE is positively associated with other proinflammatory advanced glycation end products (AGEs) [9] and negatively associated with other proinflammatory markers such as C-reactive protein (CRP), fibrinogen, and white blood cell counts [10].

More is known about transmembrane RAGE. Over-expression of transmembrane RAGE has been shown to have a protective role in experimental models including RSV infection in HEK293 cells [11]. Mice overexpressing *AGER*, the gene encoding RAGE, develop emphysema [12]. *AGER* knockout mice are resistant to tobacco smoke-induced lung disease [13] and are protected from LPS-induced lung injury [6]. Engagement of RAGE by AGEs activates inflammatory signalling pathways, including nuclear factor (NF)-kB [14] and several mitogen-activated protein kinases [15, 16]. This RAGE signalling may contribute to the sustained inflammation seen in COPD. When the extracellular portion of RAGE is cleaved, the protein becomes soluble (sRAGE) and can be measured in serum and plasma. sRAGE has been hypothesized to bind competitively to AGEs, thus reducing the transmembrane signalling of RAGE and of other pathogen-associated molecular patterns (PAMPs) or damage-associated molecular pattern (DAMPs) receptors through other pattern recognition receptors (PRRs) implicated in chronic lung inflammation [17].

In human studies, four large COPD cohorts (ECLIPSE, COPDGene, TESRA, SPIROMICS) [18–20] and several smaller studies [21–23] have reported that sRAGE is the biomarker showing the strongest known association with emphysema, even independent of airflow obstruction and other clinical covariates (age, sex, current smoking, pack-years, BMI, and prior exacerbation history). All studies demonstrated that lower levels of plasma or serum sRAGE were associated with more emphysema as measured by the 15th percentile density of lung density (PD15) or the low attenuation area at -950 Hounsfield units (LAA, the percent lung tissue voxels less than -950 HU). One study (ECLIPSE) was sufficiently powered to show that lower sRAGE was associated with more rapid progression of emphysema as measured by change in PD15 over time [20]. Although abundant evidence supports a cross-sectional association between plasma/serum sRAGE and emphysema/airflow obstruction, there are few reports of its association with COPD progression, its receiver operating characteristics, and how genetics simultaneously impacts protein level and disease associations. The goal of this study is to conduct all of these evaluations using 4 different COPD cohorts and summarize results using a meta analysis for sRAGE’s association with PD15_adj._ and FEV_1_ decline.

## Methods

### Cohorts

This analysis includes data from participants from four independent cohorts: Evaluation of COPD Longitudinally to Identify Predictive Surrogate End-points (ECLIPSE) [24]; Genetic Epidemiology of COPD (COPDGene) [25]; Subpopulations and Intermediate Outcome Measures in COPD Study (SPIROMICS) [26]; and Specialized Center for Clinically Oriented Research (SCCOR) in COPD at the University of Pittsburgh [27, 28]. Although all four cohorts enrolled predominantly older current and former smokers, there were some differences in study recruitment and the percentage of participants with COPD. COPDGene and SPIROMICS were multi-center U.S. cohorts of current and ex-smokers (> 10 and 20 pack-years respectively) with and without COPD. ECLIPSE was an international cohort from 12 countries that included predominantly moderate-very severe COPD subjects. The Pittsburgh COPD SCCOR was a single-center U.S. study. All participants signed a written informed consent. All studies were approved by the ethics and review boards at all participating centers. The current analyses include only the subset of subjects from those four cohorts who had at least one measurement of sRAGE and either spirometry or quantitative CT measurements of emphysema (Table 1).

**Table 1:** Baseline clinical characteristics of subjects who have an sRAGE measurement by cohort

	COPDGene (n = 1443)	ECLIPSE (n = 2349)	SCCOR (n = 399)	SPIROMICS (n = 1623)	p-value
Age (mean ± SD)	61 ± 9	62 ± 8	65 ± 6	64 ± 9	< 0.001
Sex (male) (%)	49%	62%	53%	54%	< 0.001
Race (%)					
Non-Hispanic White	86%	93.5%	95%	75%	< 0.001
Non-Hispanic African American	14%	1.5%	4%	16%	
Other	0%	5%	1%	9%	
BMI (kg/m^2^) (mean ± SD)	29 ± 6	27 ± 5	28 ± 4	28 ± 5	< 0.001
Never Smoker (%)	2%	9%	0%	8%	< 0.001
Current Smoker (%)	39%	35%	42%	34%	
Pack-years median (5th and 95th percentile)	38.4 (11.3; 90.0)	39 (0; 95)	46.0 (19.0; 118.0)	42.0 (0; 96.0)	< 0.001
COPD (%)	41%	78%	48%	62%	< 0.001
PRISm (%)	10%	0.2%	5%	2%	< 0.001
FEV_1_ (% predicted) (mean ± SD)	81 ± 25	62 ± 30	83 ± 20	76 ± 26	< 0.001
FEV_1_ (L) (mean ± SD)	2.36 ± 0.91	1.79 ± 1.00	2.39 ± 0.76	2.15 ± 0.91	< 0.001
FVC (L) (mean ± SD)	3.44 ± 1.00	3.30 ± 1.03	3.54 ± 0.89	3.44 ± 1.02	0.006
Emphysema (% LAA < − 950 HU) median (5th and 95th percentile)	1.40 (0.08; 26.19)	11.48 (0.49; 39.18)	0.80 (0.10; 17.40)	3.07 (0.29; 29.54)	< 0.001
PD15_adj_ (g/L) (mean ± SD)	89 ± 24	61 ± 26	87 ± 21	83 ± 26	< 0.001
History of diabetes (%)	11%	9%	8%	13%	< 0.001
History of heart attack (%)	6%	8%	5%	6%	0.009
History of coronary artery disease (%)	7%	NC	6%	9%	0.12
History of stroke (%)	2%	3%	3%	4%	0.11
Follow-up (years) median (5th and 95th percentile)	5.14 (0; 10.1)	3.0 (1.5; 3.0)	6.0 (2.0; 6.0)	3.1 (0; 7.5)	< 0.001
Percentage of visits with a spirometry per participant					
0	0.1%			0.1%	
1	39%	0.04%		12%	
2	44%	0.04%	39%	12%	
3	16%	1%	61%	18%	
4		4%		34%	
5		4%		24%	
6		5%			
7		10%			
8		76%			
Number of visits with CT scan per participant					
0	1%	7%		0.1%	
1	41%	11%		13%	
2	58%	21%	39%	50%	
3		61%	61%	36%	

To evaluate differences between cohorts, analysis of variance (ANOVA) was used for normally distributed continuous variables and Kruskal–Wallis test for non-normaly distributed variables; and a Chi square/Fisher’s exact test for categorical

*COPD* chronic obstructive pulmonary disease, *PRISm* Preserved Ratio Impaired Spirometry, *FEV*_*1*_ forced expiratory volume in one second, *FVC* forced vital capacity, *PD15*_*adj*_ HU of the 15th Percentile adjusted for total lung capacity, *NC* not collected

**Fig. 1:**
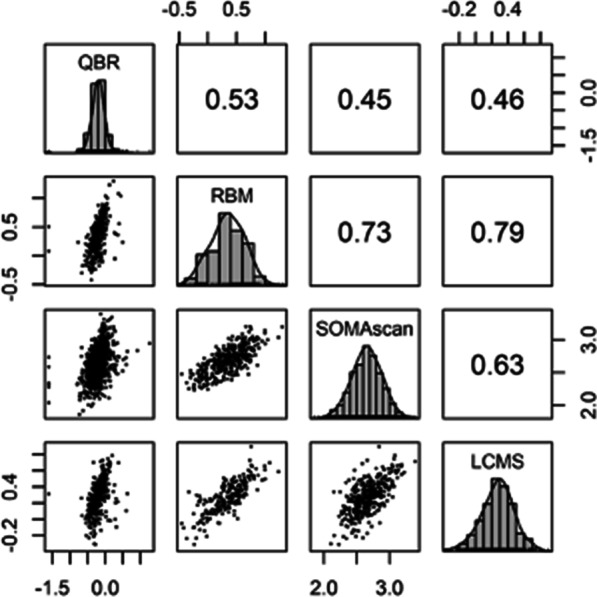
sRAGE correlation among different platforms: Quotient Bioresearch (QBR, n = 1448), Rules Based Medicine (RBM, n = 594), SOMAscan (aptamer 4125_52_2, n = 1248) and liquid chromatography/mass spectrometry (LCMS, n = 509). Axes are on a log_10_ scale. Units are ng/ml (QBR, RBM, LCMS), and scale free (SOMAscan). Data are from the COPDGene cohort. p-value < 0.001 for all correlations shown

*COPDGene (NCT02445183)* enrolled 10,300 subjects ages 45–80. sRAGE was measured in a representative sample of 1,443 subjects at the baseline using fresh frozen plasma with an sRAGE assay by Quotient Bioresearch (QBR) as previously described [29]. Additional sRAGE assays were performed in 594 subjects using fresh frozen plasma, with an sRAGE assay by Myriad-Rules Based Medicine (Myriad-RBM) as previously described [30], in 509 subjects using liquid chromatography-mass spectrometry (LCMS) [31], and in 1243 subjects using an sRAGE specific aptameric assay (Sequence ID 4125_52_2) on the SOMAscan 1.3 K panel (Fig. 1). Spirometry and CT scans were obtained at baseline and Year 5, with spirometry data available for the first 2,088 returning Year 10 participants.

*SPIROMICS (NCT01969344)* enrolled 2973 subjects. sRAGE was measured in 1623 subjects at baseline using fresh frozen plasma from EDTA (BD) tubes with the Myriad-RBM assay, as previously described [30]. Spirometry was measured at baseline (visit 1), visit 2–4 (Year 1–3), and visit 5 (mean 6 years after baseline), and; CT scans were obtained at baseline and visit 1 (Year 1) and visit 5 (mean 6 years after baseline).

*ECLIPSE (NCT00292552)* enrolled 2746 subjects. sRAGE was measured in serum from the Year 1 visit in 2349 subjects using a QBR assay as previously described [19]. CT scans were obtained at baseline, Year 1 and Year 3. There have been previous publications of the associations between emphysema and sRAGE [20]. Spirometry was measured at baseline, 3 and 6 months, and every 6 months, with the last measurement obtained at Year 3.

*Pittsburgh SCCOR* recruited subjects primarily from the Pittsburgh Lung Screening Study cohort, a tobacco-exposed cohort with only a subset of subjects having spirometrically confirmed obstructive lung disease. The complete description of subject recruitment and clinical evaluation were described in detail elsewhere [27, 28]. A total of 399 of the Pittsburgh SCCOR subjects with available follow-up study were used to analyze sRAGE levels using ELISA (DuoSet for human sRAGE, R & D Systems) and citrate plasma according to the manufacturer’s instructions. All samples were analyzed in duplicate. Spirometry and CTs were measured at baseline, Years 2 and 6.

Clinical phenotypes and their harmonization, sRAGE assays, genotyping, and statistical analyses plan are described in the Additional file 1: Methods.

## Results

### Correlation of different sRAGE assays

Among the four platforms used to measure the same samples in COPDGene, correlations were highest between RBM and LCMS (0.79), then RBM and SOMAscan (0.73), SOMAscan and LCMS (0.63), QBR and RBM (0.53), QBR and LCMS (0.46) and lowest for QBR and SOMAscan (0.45) (Fig. 1). Bland–Altman plots reveal that there were significant differences among the means, and also proportional bias, particularly when the RBM platform was a comparator (Additional file 1: Figure S1). For this reason, we chose to meta-analysis and recommend assay specific parameters be used.

### Demographics

Baseline characteristics of the COPDGene, ECLIPSE, Pittsburgh SCCOR, and SPIROMICS cohorts are shown in Table 1. SPIROMICS and COPDGene had more than 10% minorities (mostly African Americans), but the ECLIPSE and SCCOR cohorts were almost exclusively white. The COPDGene subsets of subjects who had sRAGE measured on more than one biomarker platform were similar (Additional file 1: Table S1).

On cross-sectional analysis (Additional file 1: Table S2), higher sRAGE was significantly associated with more advanced age, female sex, and non-Hispanic white race (compared to non-Hispanic African American race). Current smoking was associated with significantly higher levels of sRAGE in ECLIPSE and SCCOR. These two cohort populations were predominantly non-Hispanic white, which is associated with higher levels of sRAGE compared to non-Hispanic African Americans. Controlling for race in the analysis of current smoking had minimal effect on the associations with current smoking in ECLIPSE (β = 0.031; p = 0.0004) or SCCOR (β = 0.059; p = 0.0082); but in COPDGene and SPIROMICS significantly higher levels of sRAGE were associated with current smoking, (β = 0.039; p = 0.001) and (β = 0.051, p = 0.0043) respectively. sRAGE was not associated with comorbidities such as diabetes, cardiovascular disease, or stroke (Additional file 1: Table S2).

### sRAGE is strongly associated with severe airflow obstruction

**Fig. 2:**
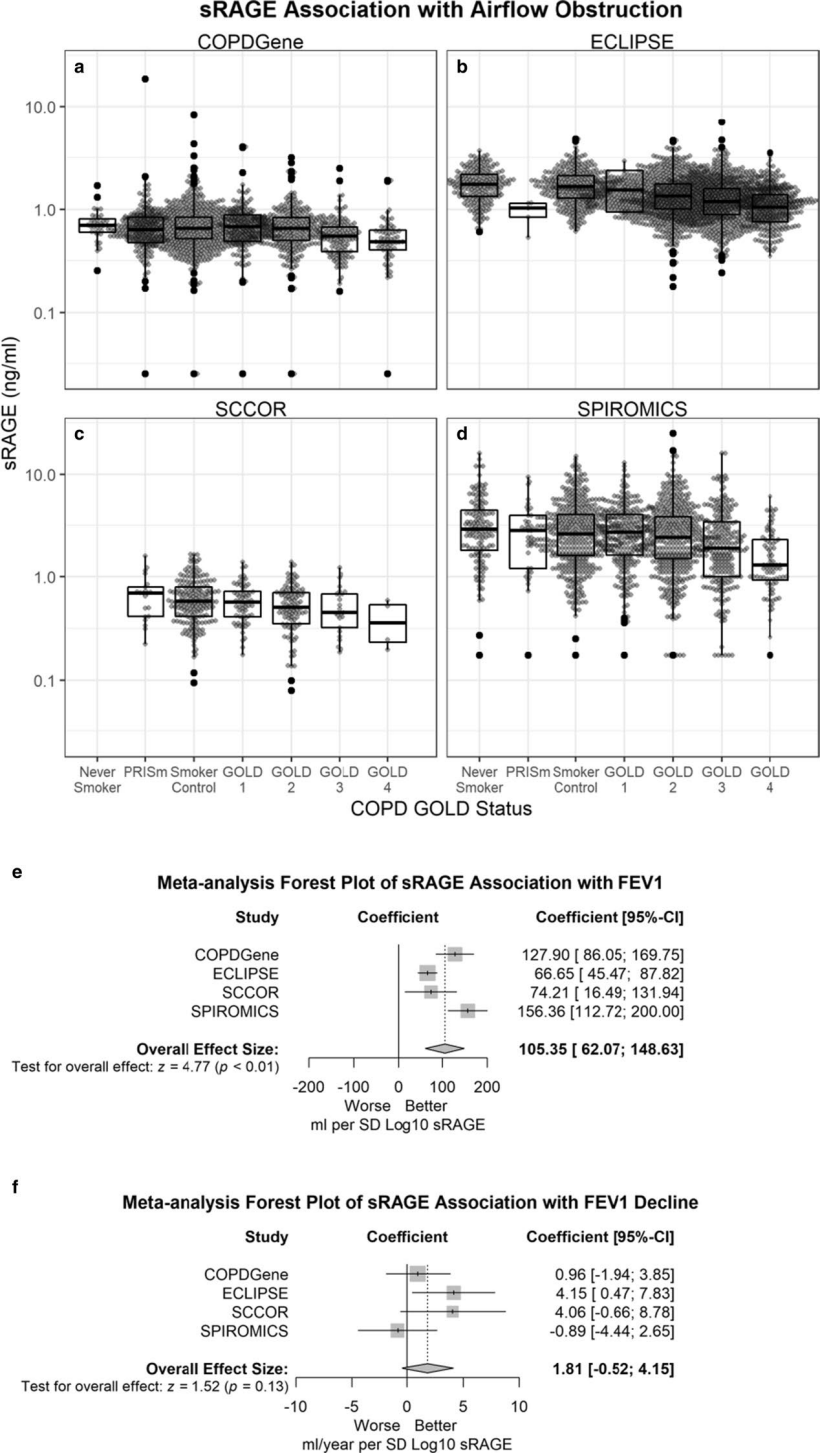
More severe airflow obstruction is associated with lower plasma and serum sRAGE in multiple cohorts and with different assay platforms for sRAGE. sRAGE is shown on the log-scale y-axis. Shown are the QBR assays for COPDGene (n = 1437) (**a**) and ECLIPSE (n = 2342) (**b**), DuoSet for sRAGE assay for SCCOR (n = 399) (**c**), and RBM assay for R&D SPIROMICS (n = 1620) (**d**). Median, 25th percentile, 75th percentile, and whiskers (the minimum of 1.5 times interquartile range (IQR) or highest/lowest value) are shown in the box plots. **e** Forest plot of sRAGE effect size estimates for baseline FEV_1_ for each cohort (squares) as well as a weighted estimate of the meta-analysis (diamond). The shaded represents the interquartile range and the whiskers represent the 95% confidence interval. **f** Forest plot of sRAGE effect size estimates with FEV_1_ decline for each cohort (squares) as well as a weighted estimate of the meta-analysis (diamond). The shaded represents the interquartile range and the whiskers represent the 95% confidence interval

sRAGE was significantly lower in subjects with airflow obstruction compared to never smokers and current and former smokers without COPD (Fig. 2; P < 0.001 for all cohorts except SCCOR: P = 0.03) and with adjusted decrease in FEV_1_ at baseline (Table 2). After adjustment for covariates, one standard deviation lower log_10_ sRAGE was associated with a weighted average of 105.35 ml lower FEV_1_ (62.07; 148.63) (Fig. 2). After adjustment for covariates, sRAGE was not significantly associated with changes in FEV_1_ over time in 3 cohorts, except in ECLIPSE (Fig. 2).

**Table 2:** Results from the random coefficient models for change in FEV_1_, (coefficients per standard deviation of Log_10_ sRAGE)

Cohort	Effect on baseline FEV_1_ (ml per SD Log_10_ sRAGE)		Effect on annual change in FEV_1_ (ml/year per SD Log_10_ sRAGE)	
	Coefficient (SE)	p-value	Coefficient (SE)	p-value
COPDGene QBR (n = 1408)	127.90 (21.35)	< 0.0001	0.96 (1.48)	0.52
ECLIPSE (n = 1847)	66.65 (10.80)	< 0.0001	4.15 (1.88)	0.0272
SCCOR (n = 399)	74.21 (29.45)	0.0121	4.06 (2.41)	0.09
SPIROMICS (n = 1408)	156.36 (22.27)	< 0.0001	− 0.89 (1.81)	0.62

### sRAGE is associated with the presence and severity of emphysema, but not progression of emphysema

**Fig. 3:**
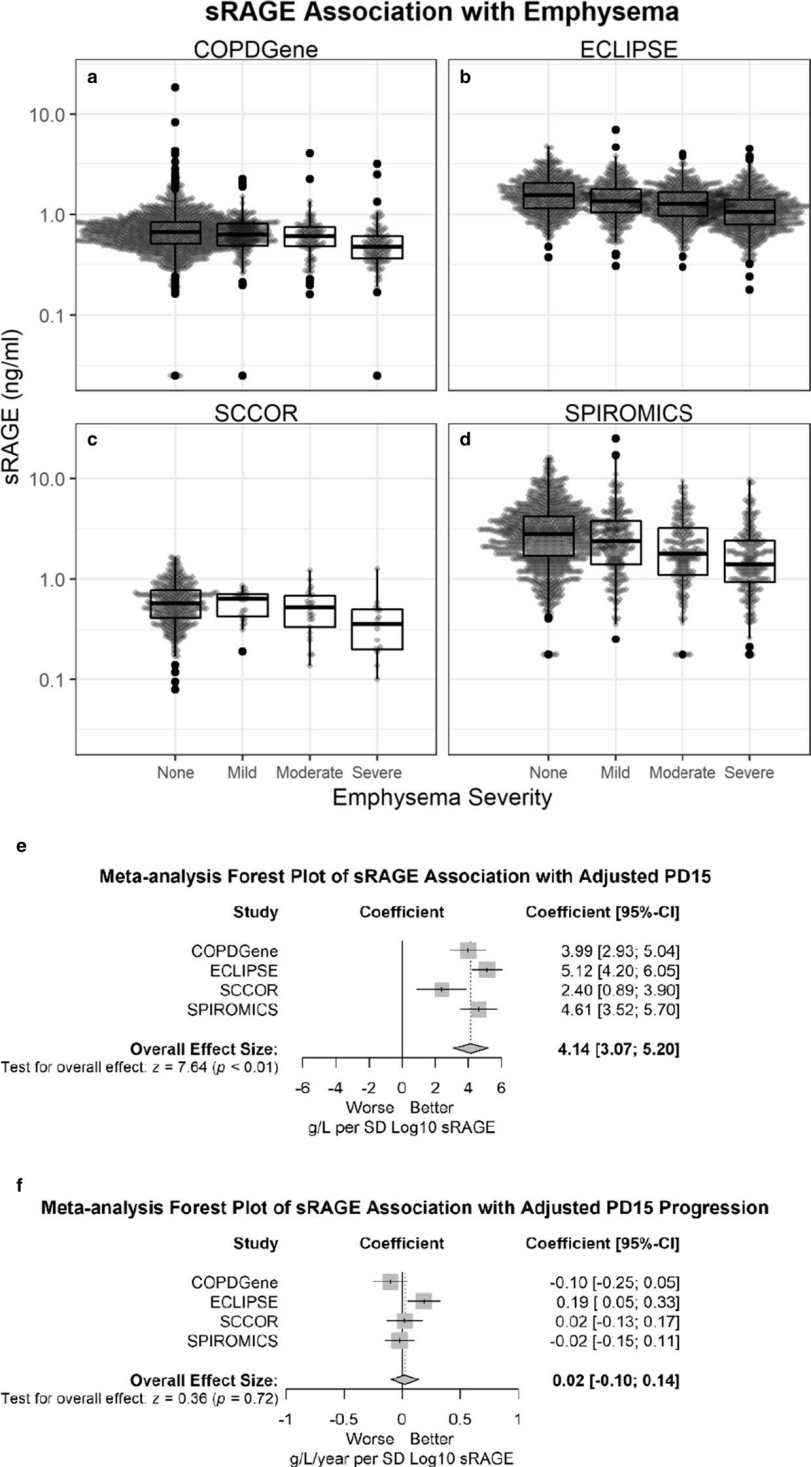
Severe emphysema is associated with lower sRAGE. sRAGE is shown on a log-scale y-axis. Each dot represents one subject. Overall p-value < 0.001 for all cohorts [COPDGene (n = 1372) (**a**), ECLIPSE (n = 1849) (**b**), SCCOR (n = 399) (**c**), and SPIROMICS (n = 1477) (**d**)]. Emphysema severity was defined as (LAA ≤ 5%), mild (LAA > 5 and ≤ 10%), moderate (LAA > 10 and ≤ 20%), or severe (LAA > 20%). Median, 25th percentile, 75th percentile, and whiskers (the minimum of 1.5 times IQR or highest/lowest value) are shown in the box plots. **e** Forest plot of sRAGE effect size estimates for baseline PD15_adj_ for each cohort (squares) as well as a weighted estimate of the meta-analysis (diamond). The shaded represents the interquartile range and the whiskers represent the 95% confidence interval. **f** Forest plot of sRAGE effect size estimates for a change in PD15_adj_ for each cohort (square) as well as a weighted estimate of the meta-analysis (diamond). The shaded represents the interquartile range and the whiskers represent the 95% confidence interval

Emphysema and more severe emphysema were associated with lower plasma or serum sRAGE in all cohorts regardless of whether emphysema was assessed by lung attenuation area below − 950 HU (p < 0.001) (Fig. 3) or PD15_adj_ (P < 0.001) (Additional file 1: Figure S2). Compared to no visual emphysema, sRAGE was significantly lower (p-value < 0.05) for moderate, confluent, and advanced destructive emphysema for COPDGene and ECLIPSE, and only for advanced destructive emphysema for SCCOR (Additional file 1: Figure S3). These associations were significant even after adjusting for important clinical predictors of emphysema, including age, sex, race, height, weight, smoking status, pack-years, exacerbations, and airflow limitation (GOLD group) (Table 3; Fig. 3). Lower sRAGE was associated with more emphysema progression in the ECLIPSE cohort, but not in COPDGene, SPIROMICS, or SCCOR (Fig. 3).

**Table 3:** Results from the random coefficient models for change in PD15_adj._, (coefficients per standard deviation of Log_10_ sRAGE)

Cohort	Effect on baseline PD15_adj_ (g/L per SD Log_10_ sRAGE)		Effect on annual change in PD15_adj_ (g/L/year per SD Log_10_ sRAGE	
	Coefficient (SE)	p-value	Coefficient (SE)	p-value
COPDGene QBR (n = 1402)	3.99 (0.0.54)	< 0.0001	− 0.10 (0.08)	0.18
ECLIPSE (n = 1699)	5.12 (0.47)	< 0.0001	0.19 (0.07)	0.009
SCCOR (n = 399)	2.40 (0.77)	0.0343	0.02 (0.08)	0.81
SPIROMICS (n = 1406)	4.61 (0.56)	< 0.0001	− 0.02 (0.06)	0.75

### sRAGE receiver operating characteristic (ROC) curves for emphysema

We tested the sensitivity and specificity of sRAGE using ROC for both quantitative emphysema (Additional file 1: Figure S4) and qualitative emphysema (Additional file 1: Figure S5). The ROC area under the curve increased (COPDGene 0.68–0.73, ECLIPSE 0.67–0.68, SCCOR 0.60–0.78, SPIROMICS 0.64–0.69) as the emphysema cutoff increased from 5 to 25% (Additional file 1: Table S3). ROC estimates for sRAGE were slightly lower for visually assessed emphysema (present/absent) (COPDGene: LCMS 0.57, QBR 0.53, RBM 0.63, SOMAscan 0.56; ECLIPSE 0.54; SCCOR 0.52; SPIROMICS 0.58) (Additional file 1: Figure S5, Table S4); however, ROC were higher for DLco (Additional file 1: Figure S6, Table S5).

### Effect of rs2070600 and rs2071288 genotypes on sRAGE measured levels and interaction with clinical phenotypes

Prior studies have reported that the *AGER* rs2070600 minor allele variant genotype (A versus common allele G) was associated with lower serum sRAGE [32]. This coding variant results in the substitution of a glycine-to-serine at amino acid position 82 (G82S). Using an RBM assay for sRAGE we have reported a similar association in both COPDGene (Additional file 1: Figure S7A) and SPIROMICS (Additional file 1: Figure S7B) [30]. Similar associations have been reported in the ECLIPSE and TESRA cohorts [19]. These used the same monoclonal antibody for capture. We also found the same association using the 4125_52_2 aptamer on the SOMAscan 1.3 k assay, which is an antibody free assay that uses aptamers for protein detection, (Additional file 1: Figure S7A); however, in COPDGene using a Quotient Bioresearch (QBR) assay, we found no association between the rs2070600 genotype and plasma sRAGE (Additional file 1: Figure S7A). Since the QBR assay correlates (⍴ = 0.53; Fig. 1) with the RBM assay in the COPDGene subjects, this suggests certain platforms may not be as sensitive to the presence of the G82S variant.

In African Americans, the minor allele (A) of the rs2071288 SNP in the *AGER* gene has been reported to be associated with lower circulating levels of sRAGE [33, 34]. This SNP is an intronic SNP located at a splice site in intron 9 and has been reported to be associated with sRAGE levels [33–35]. In the non-Hispanic African American population in COPDGene, the rs2070600 SNP was removed from the GWAS data during the QC process because of SNP frequency < 0.01; however, rs2071288 was kept in the African American population (minor allele frequency (MAF) = 0.11), but not in the non-Hispanic whites (MAF = 0.005). In the non-Hispanic African American population, the rs2071288 genotype was found to be significantly associated in the QBR assay (p = 0.0002) sRAGE levels but not with SOMAscan (p = 0.195) (Additional file 1: Figure S7C).

**Fig. 4:**
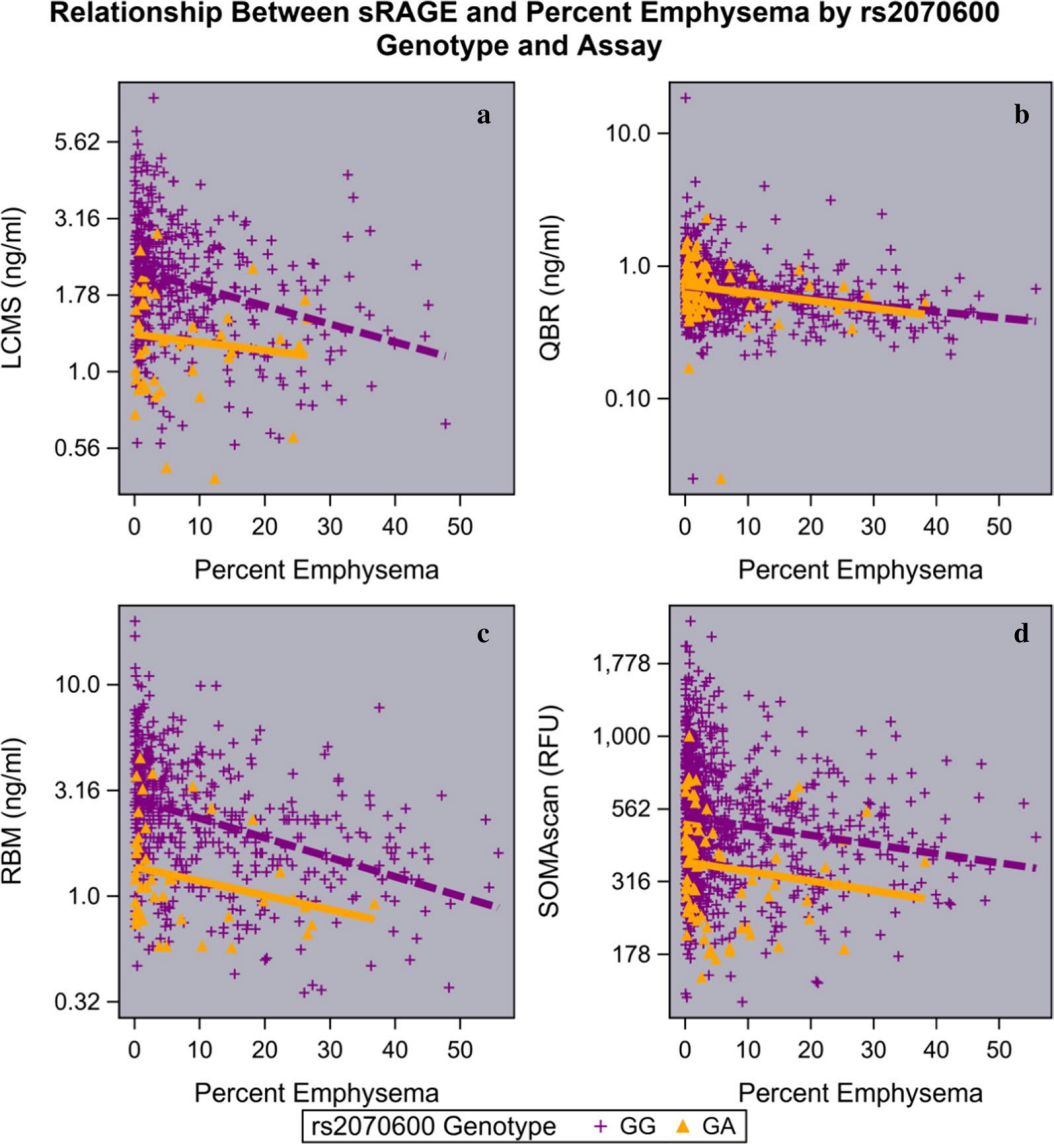
Scatter plots showing the inverse relationship between emphysema and plasma sRAGE by rs2070600 genotype showing that the slopes are not different (genotype × percent emphysema interaction) even if the intercept is lower for subjects carrying the minor allele. COPDGene subjects with sRAGE measured using **a** LCMS ( n= 491); **b** QBR (n = 1166); **c** RBM (n = 569); **d** SOMAscan (n = 998). The minor allele homozygotes are not shown because of small numbers

Although non-Hispanic whites who carry the rs2070600 variant have lower measurements of sRAGE, there is still an inverse relationship between emphysema severity and sRAGE levels regardless of genotype (Fig. 4); however, we found no significant interaction between the rs2070600 genotype and percent emphysema for sRAGE measured by either LCMS (p = 0.39), QBR assay (p = 0.70), RBM assay (p = 0.61), or SOMAscan (in regression models for emphysema (p = 0.96) for COPDGene (Fig. 4); or for ECLIPSE QBR (p = 0.65), SCCOR DuoSet (p = 0.61), and SPIROMICS RBM (p = 0.64) (Additional file 1: Figure S8A). A similar association was found with rs2071288 in non-Hispanic African Americans for QBR with an inverse relationship with a non-significant interaction between rs2071288 and percent emphysema (p = 0.51), but with the SOMAscan platform there was no inverse relationship with percent emphysema (Additional file 1: Figure S8B) and the relationship did not differ by genotype (p = 0.50).

## Discussion

This is the first report of sRAGE-COPD associations in SPIROMICS and Pittsburgh SCCOR, the first report of sRAGE associations with longitudinal decline in FEV_1_ in ECLIPSE, and the first report of an integrated protein-SNP analysis of emphysema. These observations confirm the concept that lower sRAGE is a biomarker for the presence of emphysema and airflow obstruction as have been previously reported for COPDGene and ECLIPSE [20, 29]. These associations were highly significant regardless of which sRAGE platform was used, whether plasma or serum was assayed, or how emphysema was measured (quantitative or visual). However, while baseline sRAGE was predictive of progression of emphysema and airflow obstruction in ECLIPSE [20], we were not able to replicate these associations in other cohorts. Until additional cohorts can replicate the ECLIPSE associations with emphysema progression and FEV_1_ decline, the current consensus should be limited to sRAGE serving best as a blood biomarker of emphysema/airflow obstruction or COPD affection status.

The replication of most associations across four independent cohorts is noteworthy for COPD, as there are few publications which consistently replicate biomarkers across multiple diverse cohorts. The challenge of replicating biomarkers of airflow decline or emphysema progression is not limited to proteomic approaches, but also other omics such as genetics, transcriptomics, and metabolomics. There are many genetic variants associated with lung function and COPD affection status, including the *AGER* locus which was among the first identified in large general population GWAS [36–39], and rs2070600 was recently included in a 279-SNP genetic risk score (GRS) for COPD based on a UK BioBank GWAS [36]. Since most large GWAS have only evaluated cross-sectional lung function phenotypes, there have been limited discoveries of genetic variation associated with progression of disease [40]. Furthermore, large studies of other longitudinal COPD outcomes such as exacerbations have suffered from inability to replicate significant findings across different populations [41]. The reason for replication difficulties is not completely understood, but likely includes the heterogeneity of COPD study populations, inherent variability in longitudinal spirometric and QCT measurements, as well as potentially fundamental issues such as how to define COPD affection status and how to define progression. For example, COPD affection status is based on a single spirometric measurement based on FEV_1_/FVC and severity is determined by FEV_1_% predicted. The former measure can be confounded by age and the latter may be low because full lung function was never achieved in adulthood rather than any actual loss of any lung function during adulthood. Thus, many COPD genes or biomarkers (including sRAGE) may actually be better markers of lung mass (size), density (emphysema), or structural abnormalities rather than airflow obstruction. A study that had multiple sRAGE measurements and quantitative CTs over many years (> 10) would be ideal to address this hypothesis; however, such a large studies does not yet exist.

An important aspect of this study was its use of different sRAGE assay platforms (antibody and aptamer based (SOMAscan) on the identical aliquots from the same blood sample. We demonstrated these platforms correlate with each other regardless of whether they are using antibodies or aptamers, although correlation is not perfect. We also showed that subjects who carried the minor allele for rs2070600 SNP in *AGER* (the gene that codes for RAGE) had lower measurements of sRAGE on most assay platforms. The rs2070600 SNP codes for a glycine-to-serine at amino acid 82 (G82S). Regardless of genotype, both carriers and non-carriers of the rs2070600 minor allele showed a similar inverse relationship between plasma or serum sRAGE and emphysema severity even though carriers had significantly lower sRAGE. An exception to this observation was the COPDGene QBR assay, which reportedly used a polyclonal detection antibody. There was no difference by genotype for this QBR assay, suggesting that the commonly used monoclonal or single aptamer assays may poorly bind to the G82S isoform due to epitope differences in the antibody or aptamer binding area adjacent to G82S. The G82S variant has an amino acid change adjacent to an important glycosylation site at amino acid N81. Both the G82S isoform and de-glycosylation at N81 decrease binding of RAGE to damage-associated molecular pattern (DAMPs) [42]. Additional molecular work needs to be done to determine whether the G82S isoform glycosylation pattern is sufficient to alter antigenicity of RAGE thereby leading to different binding affinities of monoclonal antibodies or aptamers. Nevertheless, the lower levels of measurement in the G82S carriers suggest that researchers consider adding rs2070600 genotype when modelling sRAGE—clinical phenotype relationships and also underscores the finding that most proteins have some genetic variants associated with their measurements and the gene-biomarker-disease modelling should account for this relationship [43].

Additionally, we were able to evaluate rs2071288, another SNP in the *AGER* gene, which is associated with circulating levels of sRAGE in non-Hispanic African American populations [33–35]. We confirmed that the minor allele (A) was associated with lower sRAGE with the QBR platform and observed a similar, but statistically non-significant, trend with the SOMAscan platform. This SNP is intronic, located at a splice site in intron 9 and is reported to be associated with diffusing capacity of carbon monoxide and with emphysema severity in COPD patients [19, 35]. This SNP has low MAF in non-Hispanic whites and our non-Hispanic African American population was a small sample size, but our findings demonstrate the importance of conducting biomarker research in ethnically and racially diverse populations to identify ethnic and racial specific gene-by-biomarker interactions.

While this study is novel in that it evaluates sRAGE platform correlations, presents new sRAGE associations with COPD severity, identifies assay specific genetic quantitative trait loci of protein expression (pQTLs), and exhaustively evaluates disease progression from four independent cohorts, there are limitations. Foremost, there is considerable heterogeneity in cohort composition with ECLIPSE having a much larger number of participants with moderate or severe COPD and emphysema. Similarly, each cohort used slightly different CT acquisition protocols [i.e., differences in tube current exposure time product (ma × sec)], which may explain higher and noisier emphysema data. Even though these cohorts are some of the largest with sRAGE and longitudinal data, a lack of association between sRAGE and disease progression in other cohorts may be due to power. In addition, it could be due to selection bias introduced by those with the most rapid decline in FEV_1_ or progression of emphysema being less likely to follow-up, resulting in results toward the null. The ROC curves for sRAGE, which do not exceed 0.75 for emphysema, suggest that it should not be used as a sole diagnostic test to rule emphysema in or out, rather that it can be used as an enrichment measure to increase or decrease the probability that an individual has emphysema similar to how ventilation perfusion scintigraphy has been used. While we did adjust analyses for important covariates such as BMI, there were some covariates that were not available in most cohorts, but might affect sRAGE measurements (e.g., lipid measurements [44]). Finally, sRAGE is also lower in participants with idiopathic pulmonary fibrosis [45], suggesting that it may be a non-specific marker of loss of lung epithelium (similar to DLco), rather than a specific marker of emphysema.

## Conclusion

In conclusion, sRAGE is identified as one of the best blood biomarkers of emphysema and airflow obstruction which makes it a strong candidate as a Drug Development Tool for screening potential clinical trial participants for interventions assessing the impact of treatment on emphysema. Additional larger studies are needed to confirm its role in predicting progression of airflow obstruction or emphysema as well as its value as a surrogate marker for efficacy of interventions. Finally, we note that there are common racially specific pQTL SNPs (rs2070600 in non-Hispanic whites and rs2071288 in non-Hispanic African Americans) and there is potential platform isoform detection specificity (monoclonal (e.g., Quantikine) versus aptamer (SOMAscan) assays versus polyclonal sRAGE assays) which may influence interpretation of sRAGE levels. Therefore, both population genetics and assay platforms should be considered when planning to interpret clinical associations.

## Supplementary Information

**Additional file 1.** Additional methods, figures, tables.

## Data Availability

The datasets used during the current study are available from the corresponding author on reasonable request.

## Abbreviations

AGEs: Advanced glycation end products
*AGER*: Gene encoding RAGE
ARDS: Acute respiratory distress syndrome
COPD: Chronic obstructive pulmonary disease
COPDGene: Genetic Epidemiology of COPD
CRP: C-reactive protein
DAMPs: Damage-associated molecular pattern
ECLIPSE: Evaluation of COPD Longitudinally to Identify Predictive Surrogate End-points
EDTA: Ethylenediaminetetraacetic acid
ELISA: Enzyme-linked immunosorbent assay
FEV_1_: Forced expiratory volume in one second
FEV_1_/FVC: Ratio of forced expiratory volume in one second to functional vital capacity
FEV_1_% predicted: FEV_1_ percent predicted: forced expiratory volume in one second/predicted FEV_1_
GOLD: Global Initiative for Chronic obstructive Lung Disease
GRS: Genetic risk score
GWAS: Genome-wide association study
LCMS: Liquid chromatography-mass spectrometry
MAF: Minor allele frequency
(NF)-kB: Nuclear factor
PAMPs: Pathogen-associated molecular patterns
PD15_adj_: 15Th percentile density of lung density
PRRs: Pattern recognition receptors
QBR: Quotient Bioresearch
QCT: Quantitative computed tomography
RAGE: Receptor of advanced glycation end products
RBM: Myriad-RBM Myriad-Rules Based Medicine
ROC: Receiver operating characteristic
RSV: Respiratory syncytial virus
SCCOR: Specialized Center for Clinically Oriented Research
SD: Standard deviation
SNP: Single nucleotide polymorphism
SPIROMICS: Subpopulations and Intermediate Outcome Measures in COPD Study
sRAGE: Soluble advanced glycation end products
%LAA: Percent low attenuation areas below − 950 Hounsfield Units on inspiratory CT
