# Supplementary material for: Soluble receptor for advanced glycation end products (sRAGE) as a biomarker of COPD
Source: Respir Res. 2021 Apr 27;22:127. doi: 10.1186/s12931-021-01686-z (PMC8076883; doi:10.1186/s12931-021-01686-z)
Supplement: Supplementary file 1 — Additional file 1. Additional methods, figures, tables. [file 12931_2021_1686_MOESM1_ESM.docx]

**Soluble receptor for advanced glycation end products (sRAGE) as a biomarker of COPD**

Katherine A. Pratte, Jeffrey L. Curtis, Katerina Kechris, David Couper, Michael H. Cho, Edwin K. Silverman, Dawn L. DeMeo, Frank C. Sciurba, Yingze Zhang, Victor E. Ortega, Wanda K. O’Neal, Lucas A. Gillenwater, David A. Lynch, Eric A. Hoffman, John D. Newell Jr, Alejandro P. Comellas, Peter J. Castaldi, Bruce E. Miller, Simon D. Pouwels, Nick H.T. ten Hacken, Rainer Bischoff, Frank Klont, Prescott G. Woodruff, Robert Paine, R. Graham Barr, John Hoidal , Claire M. Doerschuk, Jean-Paul Charbonnier, Ruby Sung, Nicholas Locantore, John G. Yonchuk, Sean Jacobson, Ruth Tal-singer, Debbie Merrill, Russell P. Bowler

**Additional file 1**

**Methods:**

### **Clinical subtypes**

Airflow obstruction. COPD was defined by post-bronchodilator forced expiratory volume in the first second (FEV_1_) to forced vital capacity (FVC) ratio of <0.70. Smoker controls were current or former smokers without evidence of airflow limitation (FEV_1_/FVC ≥ 0.70) and FEV1 percent predicted (FEV1%) ≥ 80.0. The severity of COPD was defined by FEV_1_% and categorized as follows: mild (≥ 80%); moderate (≥ 50% and <80%); severe (≥ 30% and <50%); or very severe (<30%). COPD progression was reported as change in FEV_1_ (ml/year). Subjects with FEV_1_/FVC ≥ 0.70 and FEV_1_% < 80% predicted were defined as having Preserved Ratio Impaired Spirometry (PRISm).

Emphysema. Emphysema was defined using the percent of voxels with Hounsfield Units (HU) < −950 (%LAA) on CT. Measurements below -1000 HU were removed from the analyses. The severity of emphysema was classified as none (LAA ≤ 5%), mild (LAA 5–≤10%), moderate (LAA 10–≤20%), or severe (LAA > 20%). Emphysema was also defined using the HU of the 15th percentile adjusted for total lung capacity (PD15_adj_) determined using the lung density (g/L) adjusted for the race‐adjusted predicted TLC value versus the actual lung volumes achieved (adj. g/L), as previously described (1). Emphysema density (HU) was converted to density (g/L) by adding 1000 to its HU value. Visual assessment of emphysema was available for cohorts as described for COPDGene (2), ECLIPSE (3), and SCCOR (4), and defined as a consensus below.

| Visual Emphysema Assessment Harmonization | | | |
| --- | --- | --- | --- |
| Consensus Name | COPDGene | ECLIPSE | SCCOR |
| None | None | Not affected | None (0 points) |
| Trace | Trace | Trivial (<5%) | 0-10% (1 point) |
| Mild | Mild | Mild (5-25%) | 11% - 25% (2 points) |
| Moderate | Moderate | Moderate (26-50%) | 26% - 50% (3 points) |
| Confluent | Confluent | Severe (51-75%) | 51% - 75% (4 points) |
| Advanced Destructive | Advanced Destructive | Very Severe (>75%) | >75% (5 points) |

rs2070600 and rs2071288 Genotyping. In COPDGene, the genotype data for rs2070600 in non-Hispanic whites and rs2071288 in non-Hispanic African Americans were determined using the Illumina HumanOmniExpress Beadchip (5). The other cohorts only had genotype data for rs2070600. SPIROMICS used the Illumina OmniExpress HumanExome BeadChip for SPIROMICS (6) and ECLIPSE the Illumina’s HumanHap550 genotyping BeadChip (version 3) (7). SCCOR genotyped rs2070600 using Taqman primer and probe set from Applied Biosystems. All single nucleotide polymorphisms (SNP) were in Hardy-Weinberg equilibrium (p>0.05).

**Statistical analysis**

All statistical analyses were conducted on the former and current smokers for all cohorts. For ECLIPSE, the smoker controls were missing data on exacerbations and were excluded from the linear mixed models for both FEV_1_ and PD15_adj_. Baseline characteristics were compared between the different cohorts using an analysis of variance (ANOVA) for normally distributed continuous variables or a Kruskal-Wallis test for non-normally distributed variables, and a chi-squared or Fisher’s exact test for categorical variables.

*sRAGE.* Serum and plasma levels of sRAGE were converted to ng/ml if reported on a different quantitative scale. sRAGE values were log_10_ transformed for all analyses and graphs. For the linear mixed models, the log_10_ transformed levels were standardized by their standard deviations in their respective studies. Pearson correlations were performed to evaluate the linear relationship between the 4 different sRAGE platforms measured in the COPDGene cohort. Additionally, Bland-Altman analyses were conducted to evaluate agreement between the different assays using log_10_ transformed sRAGE values. The results are reported as the proportionally to the magnitude of measurements.

*sRAGE association with clinical covariates.* An analysis of variance (ANOVA) was used to analyze mean differences in sRAGE levels between the different groups for categorical covariates (sex, race, current smoking status, self-reported history of: diabetes, heart attack, coronary artery disease and stroke). Race was defined as self-reporting being non-Hispanic white, non-Hispanic African American, or other race. Linear regression was used to determine the relationship between sRAGE and age.

*Modelling FEV_1_ and emphysema*. Random coefficient models with both a random intercept and slope at a subject level were constructed to evaluate the effect of baseline covariates and sRAGE on outcomes such as FEV_1_ or PD15_adj_ at baseline and their rate of change over the time period of each study (8). The random slope was based on time (years) between measurements. Interaction terms between covariates and time were included in models to control for their association with change over time, and for sRAGE to estimate its association with change over time. Models for FEV_1_ included log_10_ sRAGE with baseline covariates: age, age^2^, race, height, height^2^, weight, number of exacerbations as previously described (8), sex, current smoking status, pack-years, and time between measurements with interaction terms of years with current smoking status, sex and sRAGE. For PD15_adj_ models were constructed as previously reported in (8). Given sRAGE levels significantly differed between the races, race and race*sRAGE were included in both models to control for the differences in the populations. Because rs2070600 and rs2071288 had low minor allele counts in our stratified populations we did not include them in these analyses.

*Meta-analyses of results*. A meta-analysis was conducted for FEV_1_ decline and emphysema progression using an inverse variance weighted random-effects-model. Between study variance was estimated using the restricted maximum likelihood.

*Influence of rs2070600 and rs2071288 genotype on circulating sRAGE levels and phenotype interactions*. An ANOVA was used to evaluate the least squared means differences between genotypes and sRAGE levels. To evaluate the influence rs2070600 and rs2071288 genotypes have on sRAGE’s relationship with percent emphysema, an additive genetic model was tested for the minor allele (A) of rs2070600 and rs2071288. Linear regression was used including both the genotype, percent emphysema and percent emphysema*genotype interaction term. To control for population substructure, models for COPDGene, SPIROMICS, and ECLIPSE, included ancestry-based principal components. SCCOR did not have any ancestry-principal components, but the results are in a white population.

*Sensitivity and specificity of sRAGE and DLco percent predicted to detect emphysema.* We used logistic regression to estimate the sensitivity and specificity of sRAGE and DLco percent predicted to detect emphysema. DLco percent predicted was measured at visit 2 for COPDGene. Receiver operating characteristic curve (ROC) plots and area under the curves (AUC) were estimated for the different quantitative emphysema cutpoints and for visually assessed emphysema.

SAS (9.4) was used to create the panel graphs and all analyses, with the exception of the Bland-Altman and meta-analysis. The Bland-Altman analysis was conducted in R3.6.3 with the ‘blandr’ version 0.3.1; and meta-analysis was conducted in R 3.5.2, with packages meta (version 3.5.2), and the beeswarm plots were produced with R packages ggplot2 (version 3.1.1) and ggbeeswarm (version 0.6.0).

**Supplementary Figure and Tables**

**Figures:**

| 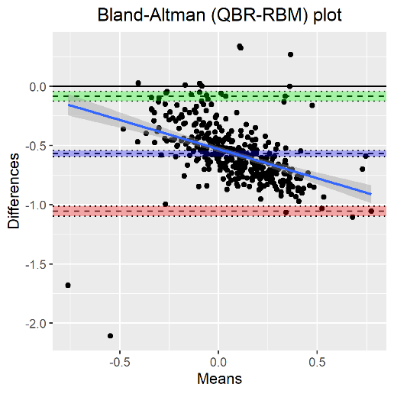 A | 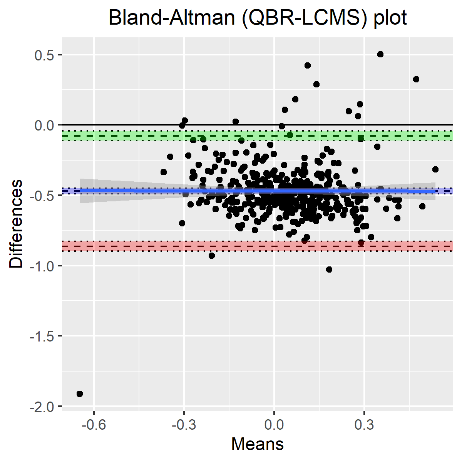 B | 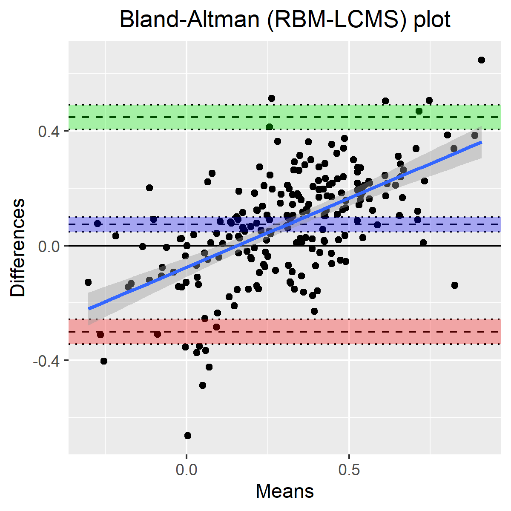 C |
| --- | --- | --- |
| 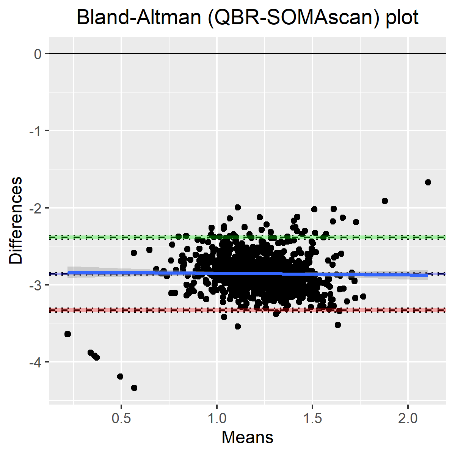 D | 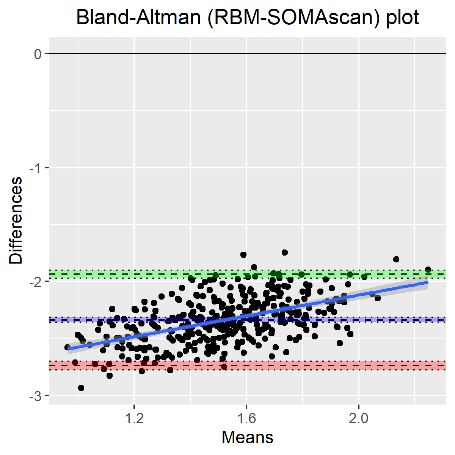 E | 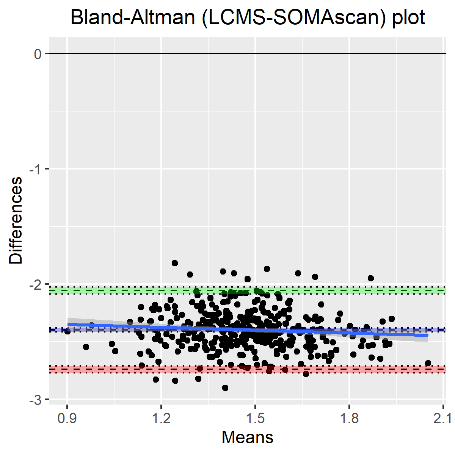 F |
| Figure S1. Bland-Altman plots between the different assays reveals significant mean differences among most of the assays and proportional bias when RBM assay was one of the comparators. Plots are the differences between: A) QBR and RBM (n=397), B) QBR and LCMS (n=384), C) RBM and LCMS (n=220), D) QBR and SOMAscan (n=1,084), E) RBM and SOMAscan (n=366), F) LCMS and SOMAscan (n=366). The middle dashed horizontal line is the bias line (the mean differences between the assays). The parallel dashed line above and below represents the 95% confidence interval (CI). The blue solid line is the regression line of the proportional differences The green, purple, blue and red shade areas represent the error margins. | | |


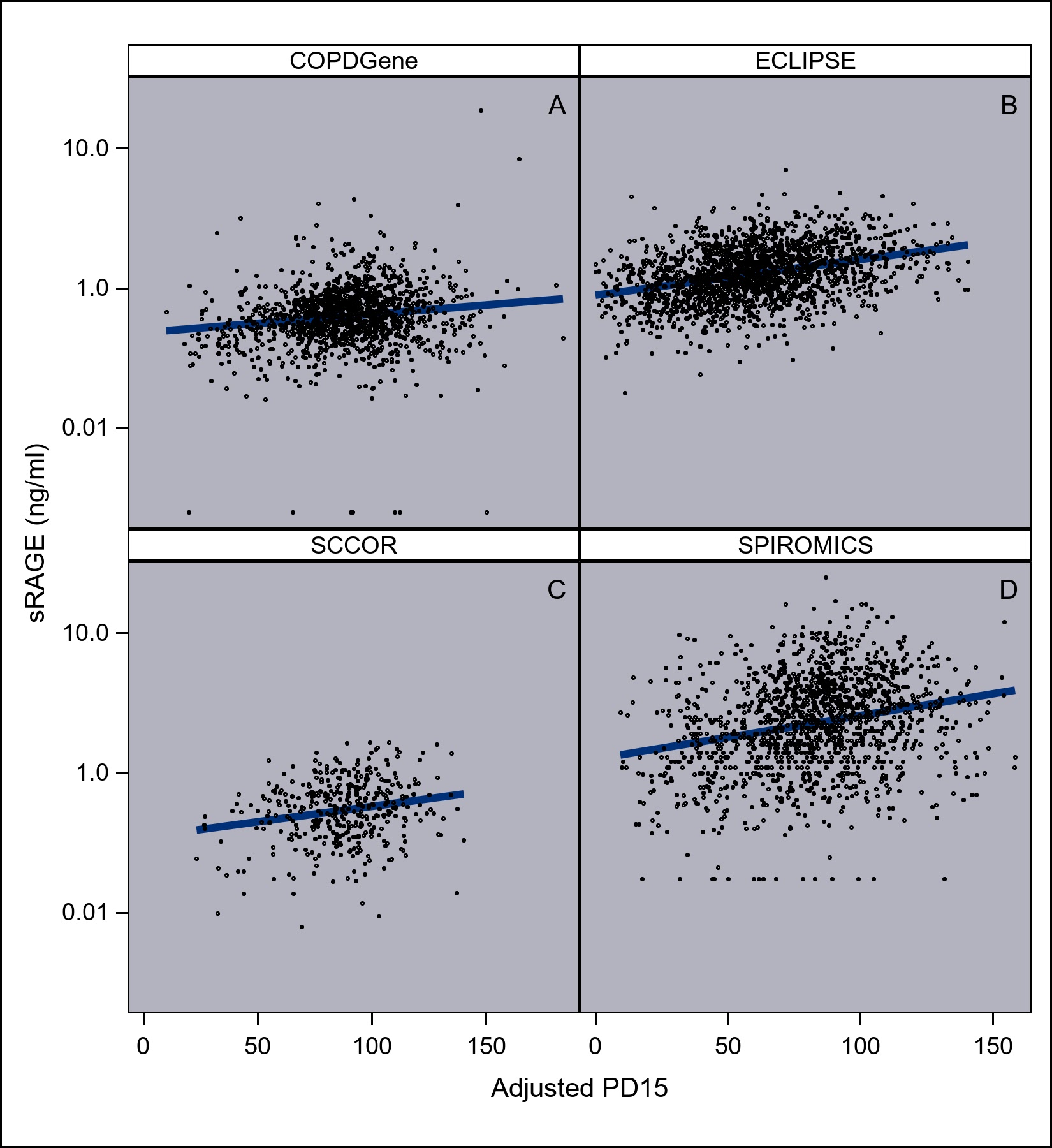


**Figure S2**: Relationship between baseline emphysema and sRAGE in smoking population. Association between sRAGE and emphysema severity as measured by the PD15_adj_ . Cohorts (platform) were (A) COPDGene (QBR, n=1,372), (B) ECLIPSE (QBR, n=1,831), (C) SCCOR (R & D DuoSet, n=399), (D) SPIROMICS (RBM, n=1,270). Each dot represents one subject. The lines represent regression lines, of which all are > 0 (p-value < 0.001).


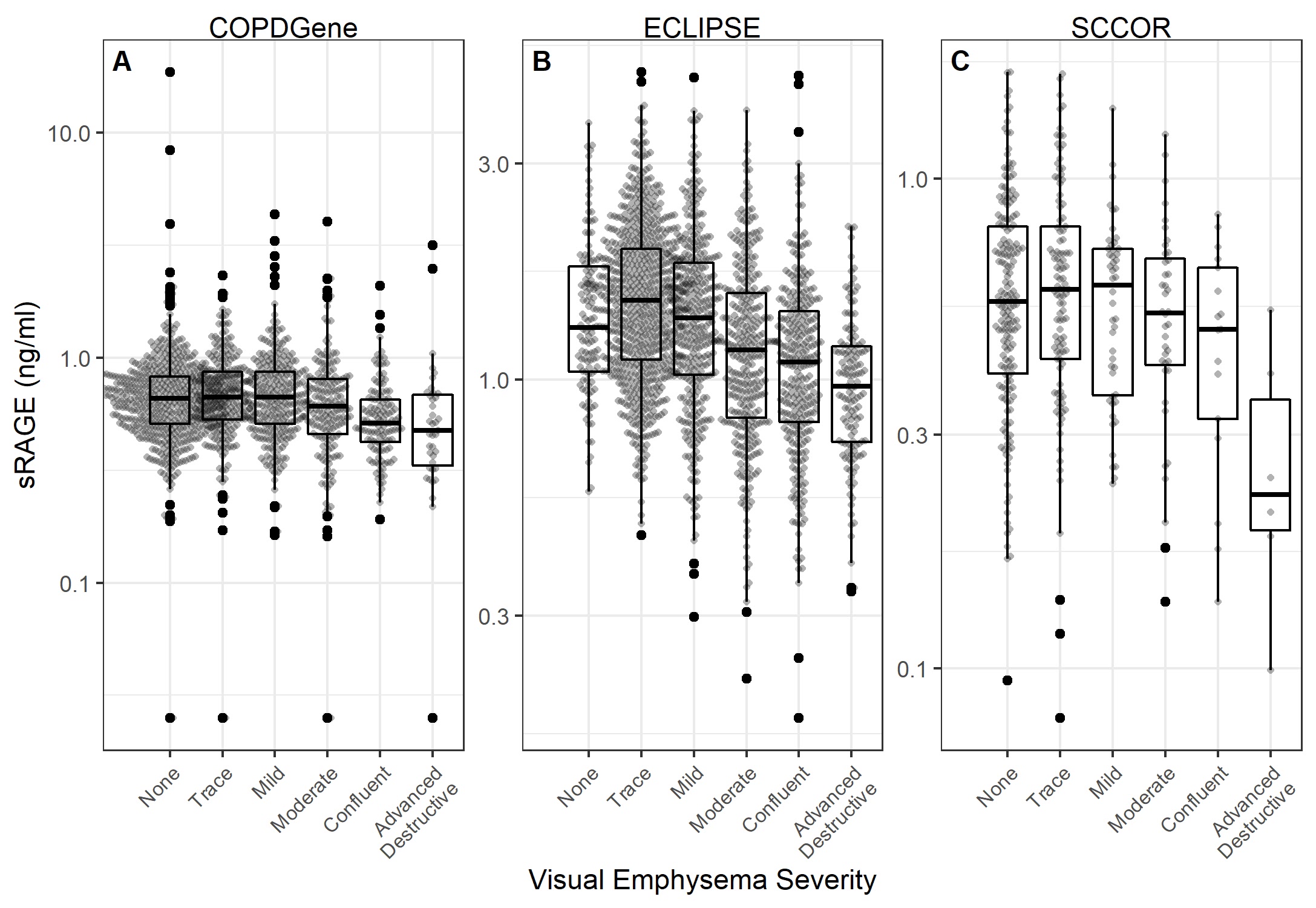


**Figure S3**: Association between sRAGE and visual emphysema severity: A) COPDGene (n=1,646), B) ECLIPSE (n=1,971), C) SCCOR (n=399). Each dot represents one subject.


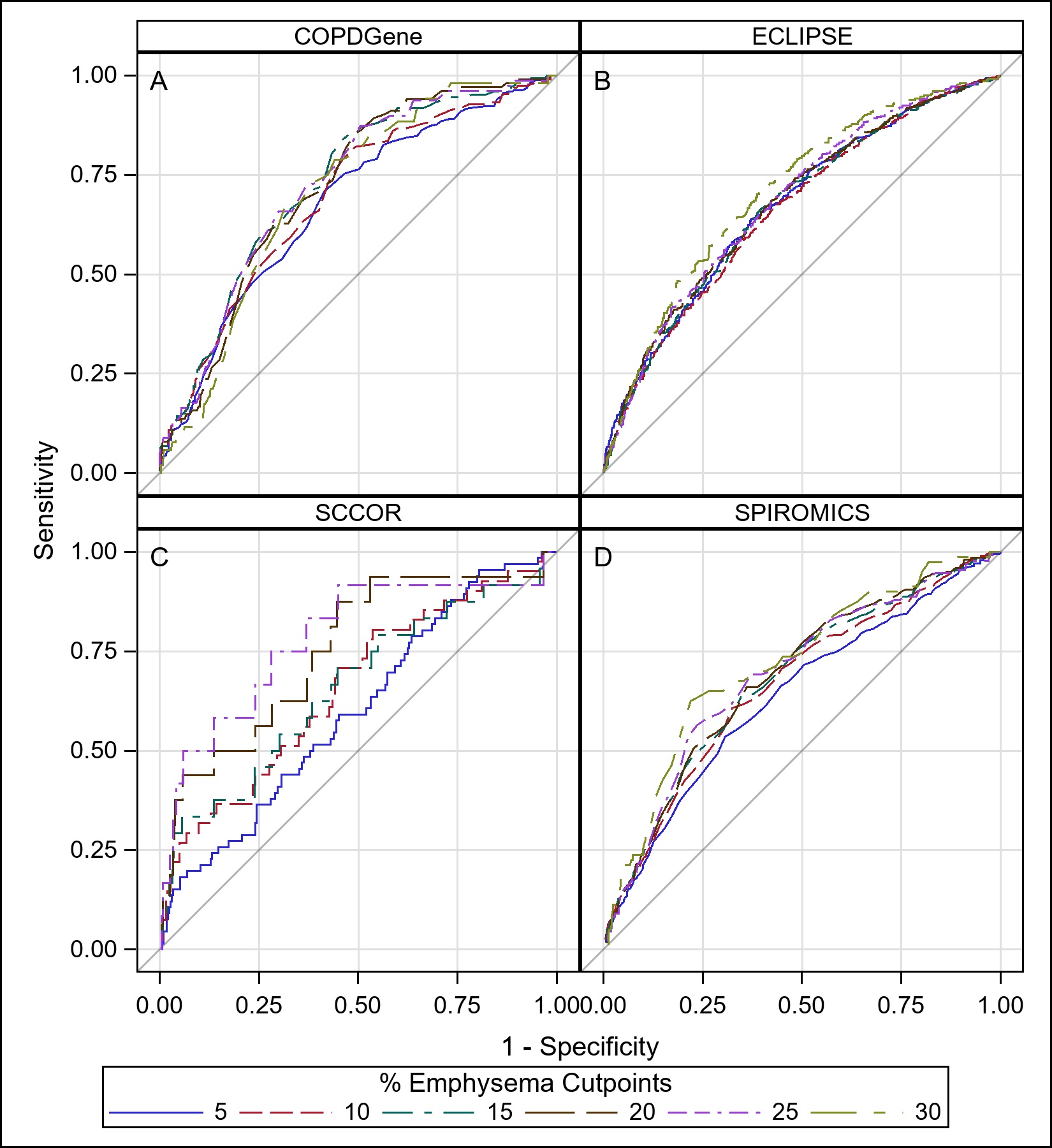


**Figure S4**. sRAGE ROC curves of the different percent emphysema cut-points. Shown are data using the RBM assay in COPDGene (n=580) (A) and SPIROMICS (n=1,477) (D), QBR assay in ECLIPSE (n=1,849) (B), and R & D DuoSet in SCCOR (n=399) (C). Different curves represent different definitions based on the %LAA < 950 Hounsfield Units.


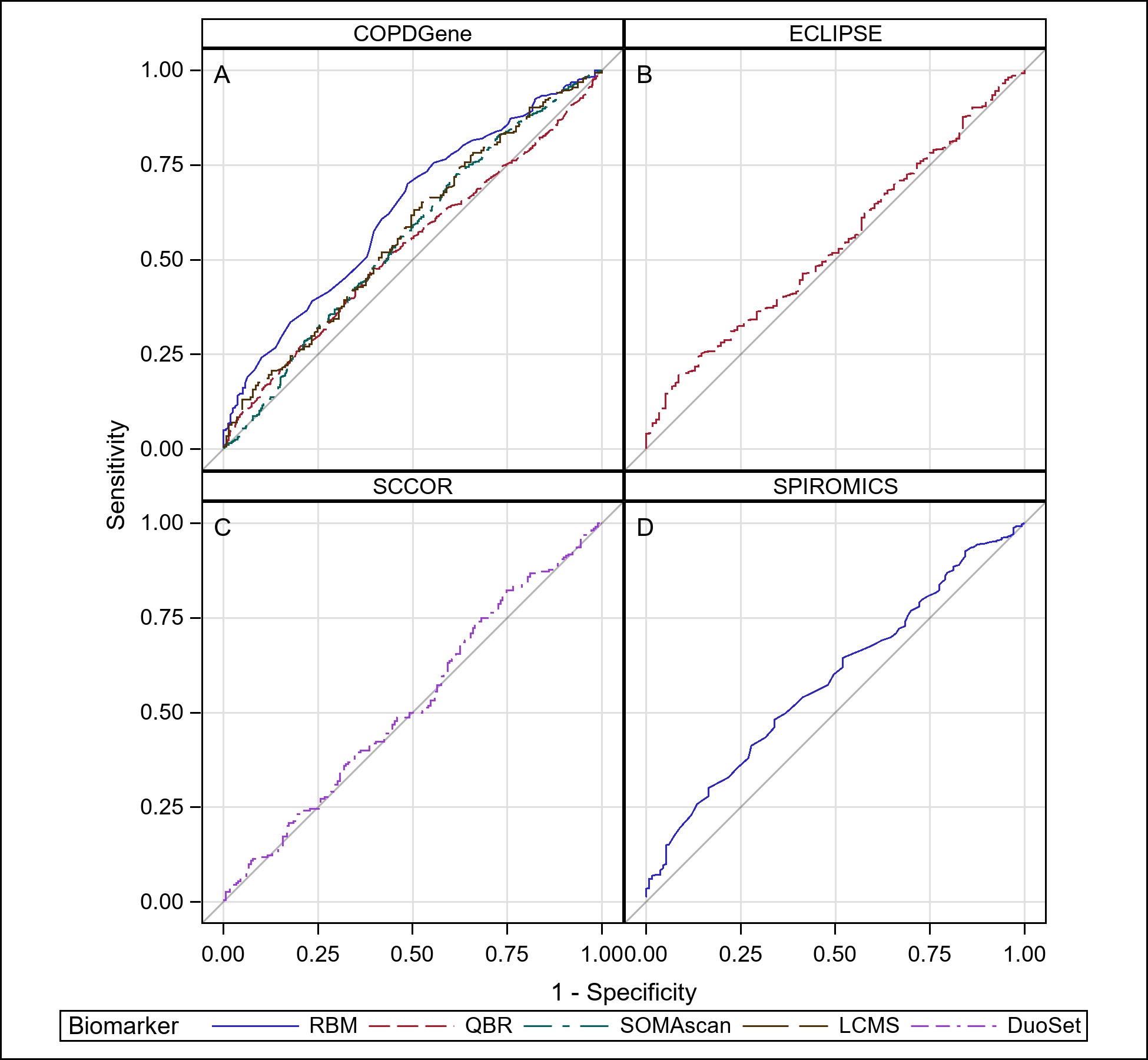


**Figure S5**. ROC curves for sRAGE and visually assessed emphysema. Shown are data using different assays in COPDGene (A), QBR assay in ECLIPSE (B), and R & D DuoSet in SCCOR (C), and RBM in SPIROMICS (D). Emphysema was assessed by readers (see methods) as yes or no.

**Figure S6**. ROC of different cutpoints of % emphysema and DLco percent predicted for all visit 2 observations with sRAGE measurements (n=4,624).


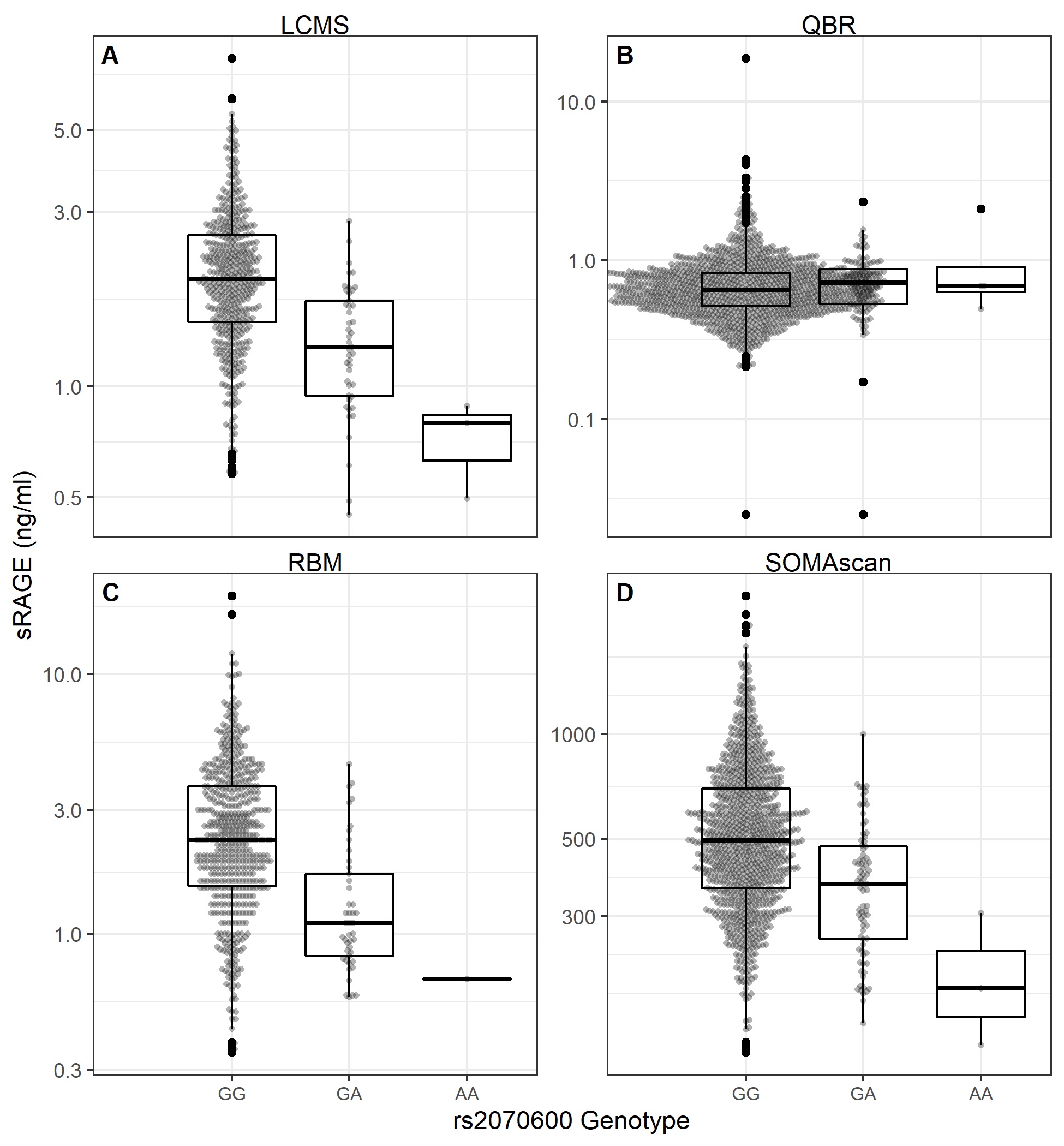


**Figure S7A**. Relationship between rs2070600 and plasma sRAGE. (A) Using LCMS assay (p-value < 0.001, n=506), (B) using QBR (P = N.S., n=1,197), (C) using RBM assay (p-value < 0.001, n=584), (D) using SOMAscan aptamer X4125_52_2 (p-value < 0.001, n=1,027). The x-axis shows genotype (minor allele A). Lines represent divisions between quartiles. Note that only non-Hispanic white subjects are shown because the rs2070600 minor allele frequencies are very low in noon-Hispanic African-American populations.


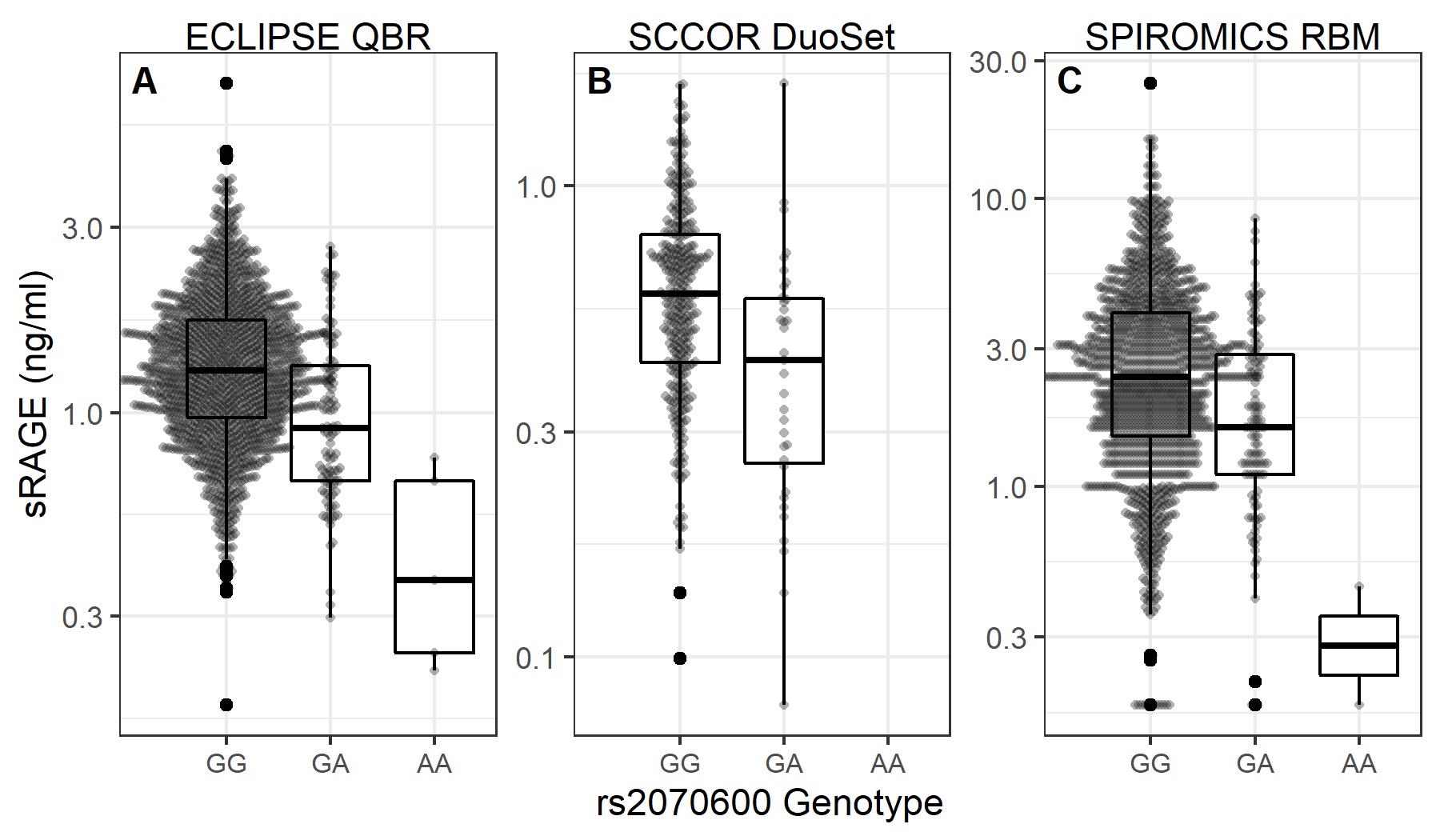


**Figure S7B.** Relationship between rs2070600 and plasma or serum sRAGE. (A) ECLIPSE using QBR (p-value <0.001, n=1,652), (B) SCCOR using DuoSet (p-value < 0.001, n=372), (C) SPIROMICS using RBM (p-value < 0.001, n=1,403). The x-axis shows genotype (minor allele A). Lines represent divisions between quartiles.


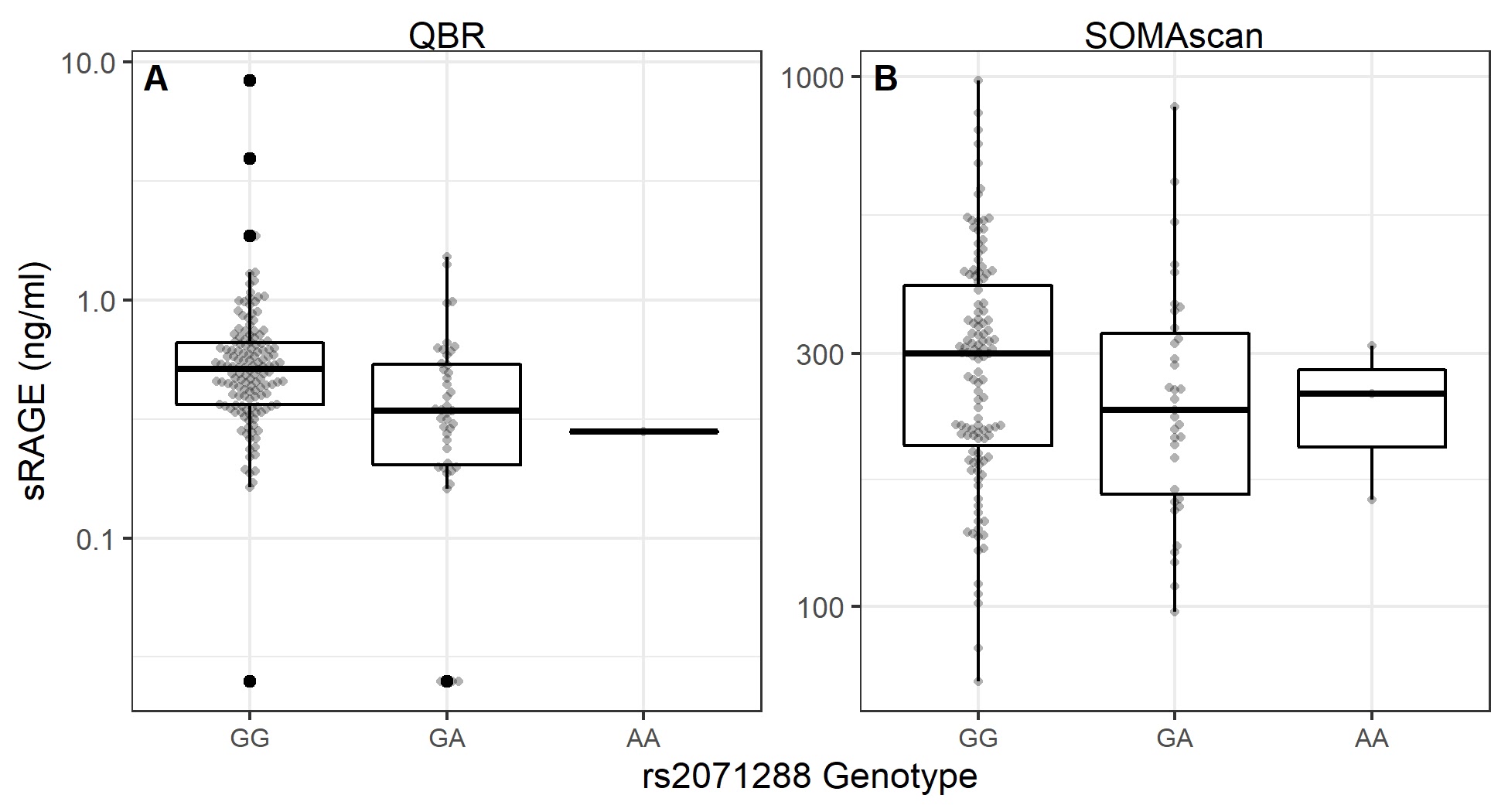


**Figure S7C.** Relationship between rs2071288 and plasma sRAGE in the COPDGene non-Hispanic African American population. sRAGE was only measured in non-Hispanic African Americans for the QBR and SOMAscan assays were: (A) QBR (p-value = 0.0002, n=188), (B) SOMAscan (p-value = 0.1950, n=157). The x-axis shows genotype (minor allele A). Lines represent divisions between quartiles.


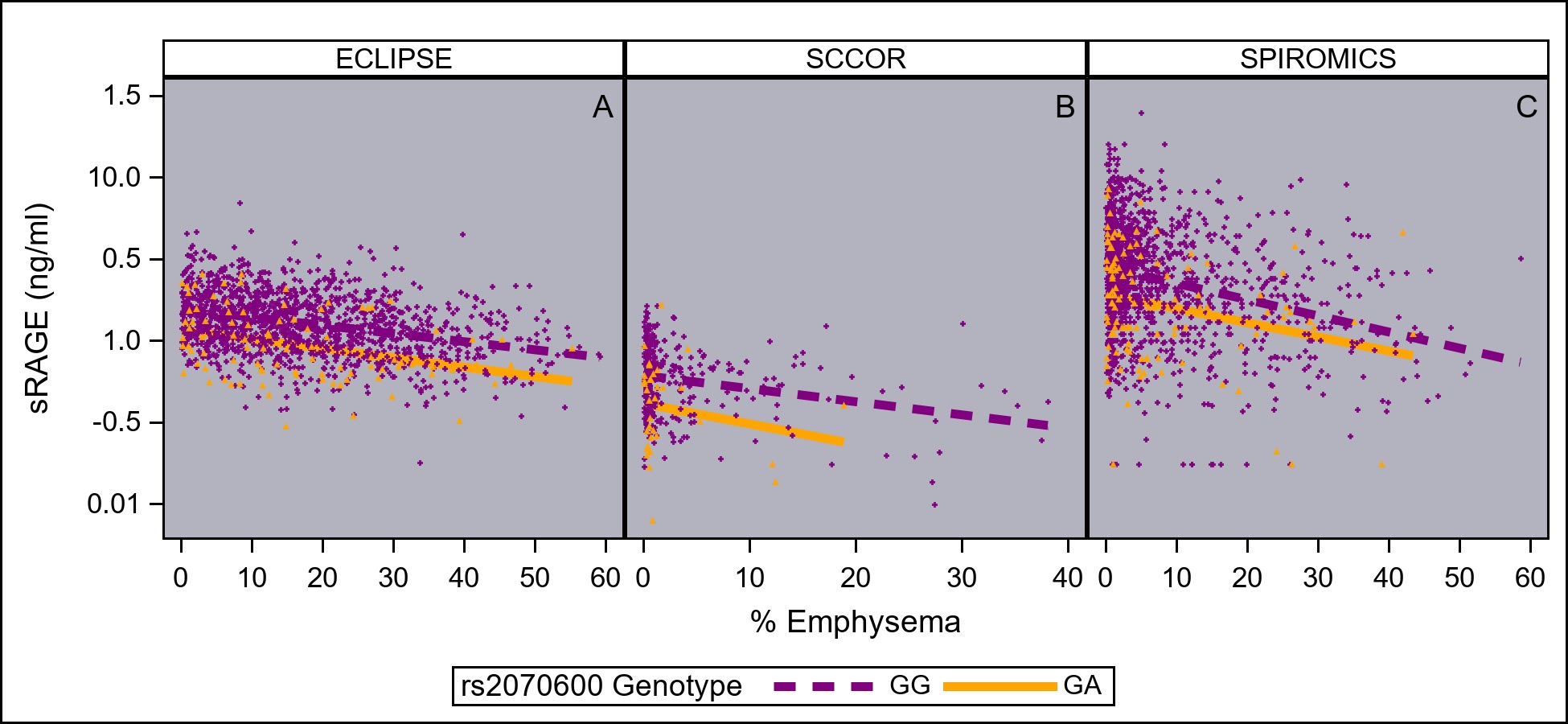


**Figure S8A**. Relationship between sRAGE and percent emphysema by rs2070600 genotype. ECLIPSE (n=1,398) (A), SCCOR (n=371) (B), and SPIROMICS (n=1,392) (C) scatter plots showing the inverse relationship between plasma sRAGE and percent emphysema by rs2070600 genotype showing that the slope is not different (genotype*percent emphysema interaction N.S.).


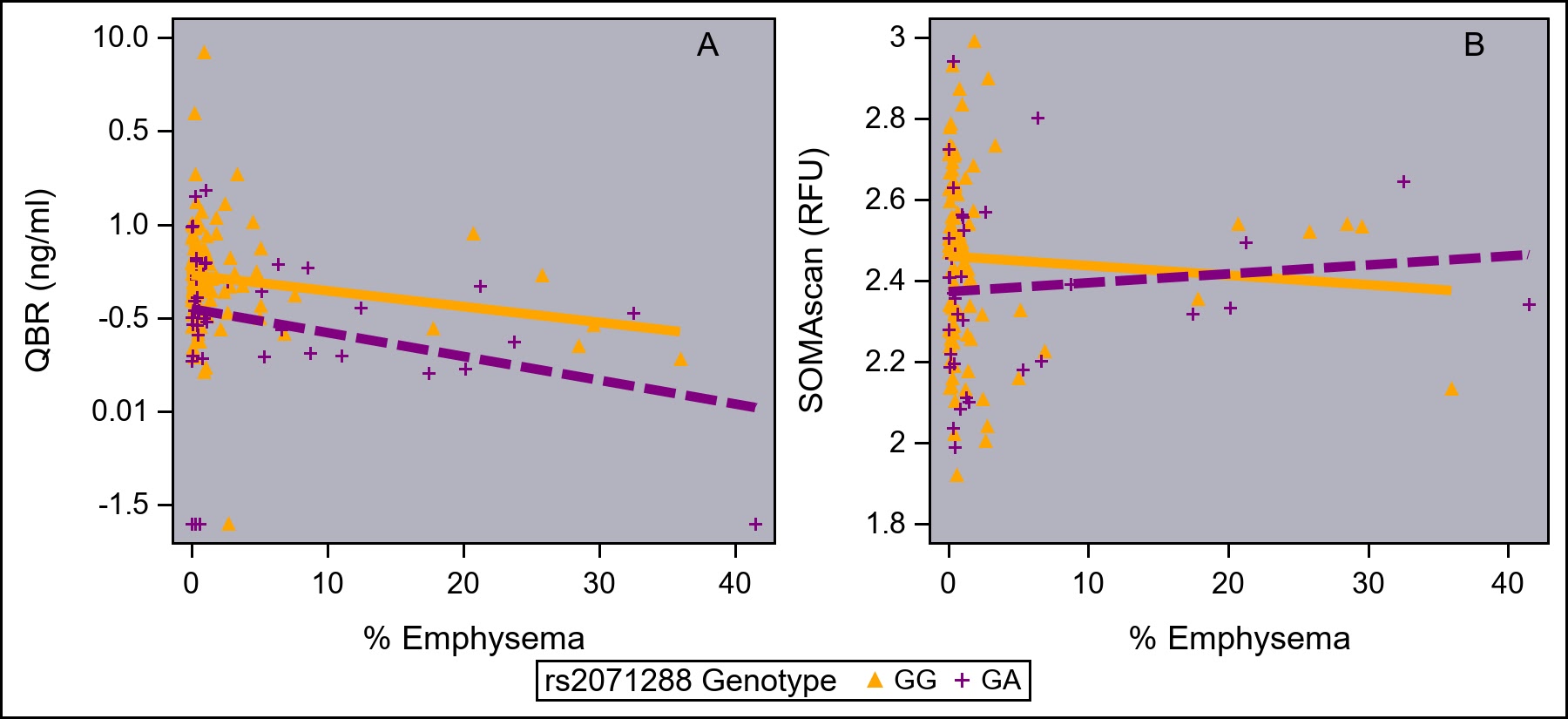


**Figure S8B**. Relationship between sRAGE and percent emphysema by rs2071288 genotype in COPDGene non-Hispanic African Americans. QBR (n=178) scatter plot (A) showing an inverse relationship between emphysema and plasma sRAGE by rs2071288 genotype and that the slope are not different (genotype*percent emphysema interaction p-value=0.51.). SOMAscan (n=147) (B) showed no relationship between percent sRAGE and percent emphysema. While the regression line for the genotype suggest an interaction there is no significant interaction between the genotypes (p=0.50). Influential and extreme outliers were found to have no effect on these results. .

**Tables:**

| **Table S1**: Baseline characteristics of COPDGene subjects who have an sRAGE measurement determined by LCMS, RBM, and SOMAscan at phase 1. | | | |
| --- | --- | --- | --- |
|  | LCMS  n=509 | RBM  N = 594 | SOMAscan  N = 1,243 |
| Age (yr) (mean ± SD) | 65 ± 7 | 64 ± 9 | 62 ± 9 |
| Sex (male) (%) | 48% | 52% | 49% |
| Race (%) |  |  |  |
| Non-Hispanic White | 100% | 99.8% | 87% |
| Non-Hispanic African American | 0% | 0.2% | 13% |
| Other | 0% | 0% | 0% |
| BMI (kg/m^2^) ( mean ± SD) | 29 ± 5 | 28 ± 6 | 29 ± 6 |
| Never Smoker (%) | 0% | 0% | 3% |
| Current Smoker (%) | 25% | 24% | 40% |
| Pack-years  median (5th and 95th percentile) | 42.0 (14.0 ; 94.0) | 42.0 (12.8 ; 98.0) | 39.4 (11.0 ; 90.8) |
| COPD Patients (%) | 64% | 58% | 45% |
| PRISm (%) | 0% | 2% | 10% |
| FEV1 (% predicted)  ( mean ± SD) | 75 ± 25 | 68 ± 30 | 78 ± 26 |
| FEV1 (L) ( mean ± SD) | 2.15 ± 0.82 | 2.02 ± 1.03 | 2.26 ± 0.91 |
| FVC (L) ( mean ± SD) | 3.42 ± 0.95 | 3.33 ± 1.03 | 3.38 ± 0.97 |
| Emphysema (% LAA < -950 HU) median (5th and 95th percentile) | 3.48 (0.17 ; 28.54) | 4.28 ( 0.14 ; 37.56) | 1.50 (0.07 ; 28.4) |
| Adjusted PD 15  ( mean ± SD) | 81 ± 23 | 76 ± 25 | 87 ± 25 |
| History of diabetes (%) | 13% | 11% | 11% |
| History of heart attack (%) | 6% | 6% | 6% |
| History of coronary artery disease (%) | 7% | 7% | 7% |
| History of stroke (%) | 1% | 2% | 3% |
| Follow-up (years)  median (5th and 95th percentile) | 5.4 (4.7 ; 10.2) | 5.2 (0 ;10.1 ) | 5.1 (0 ; 10.1) |
| Spirometries per subject: 0 |  |  | 0.2% |
| 1 | 0.4% | 36% | 40 % |
| 2 | 68.6% | 46% | 44% |
| 3 | 31% | 18% | 16% |
| CT scans per subject: 0 | 0.6% | 1% | 2% |
| 1 | 8% | 40% | 42% |
| 2 | 91% | 58% | 56% |

| **Table S2**: sRAGE associations with clinical covariates in smoking population | | | | |
| --- | --- | --- | --- | --- |
|  | COPDGene  n=1,408 | ECLIPSE  n=2,146 | SCCOR  n=399 | SPIROMICS  n=1,486 |
| Age (yr) | 0.002  (p=0.0042) | -0.0003 (p=<0.60) | 0.002  (p=0.24) | 0.0035 (p=0.004) |
| Sex (female - male) | 0.018  (p=0.13) | 0.034 (p=0.0001) | 0.055  (p=0.0146) | 0.066 (p=0.002) |
| Race (AA – NHW) | -0.168  (p<0.0001) | -0.144  (p<0.0001) | -0.223  (p<0.0001) | -0.274 (P<0.0001) |
| Race (other – NHW) | NA | -0.0008  (p=0.96) | 0.078  (p=0.48) | -0.061  (p=0.049) |
| Race (other – AA) | NA | 0.143  (p=0.0003) | 0.301  (p=0.0147) | 0.274 (p<0.0001) |
| Current Smoker | -0.003  (p=0.79) | 0.031  (p=0.0003) | 0.055  (p=0.0159) | 0.0015  (p=0.93) |
| Diabetes | -0.013  (p=0.49) | 0.027  (0.07) | -0.028  (p=0.51) | -0.0057  (p=0.83) |
| Heart Attack | 0.034  (p=0.16) | 0.029  (p=0.059) | 0.021  (p=0.70) | 0.034  (p=0.43) |
| Coronary Artery Disease | 0.022  (p=0.32) | NC | -0.092 (p=0.057) | 0.042  (p=0.18) |
| Stroke | 0.031  (p=0.42) | 0.029  (p=0.20) | -0.087  (p=0.23) | -0.0027  (p=0.95) |

Mean differences in log_10_(sRAGE) and p-value for bivariate variables and ß-estimates and p-value for continuous variables; difference in race is paired comparisons for non-Hispanic white (NHW), non-Hispanic African American (AA), and other races. NC means not collected.

| **Table S3.** ROC for the different emphysema cut-points | | | | |
| --- | --- | --- | --- | --- |
|  | Area Under the Curves | | | |
|  | COPDGene | ECLIPSE | SCCOR | SPIROMICS |
| % Emphysema cut-points | RBM  n=580 | QBR  n=1,849 | DuoSet  n=399 | RBM  n=1,477 |
| 5% | 0.675 | 0.670 | 0.599 | 0.635 |
| 10% | 0.693 | 0.660 | 0.657 | 0.663 |
| 15% | 0.727 | 0.669 | 0.657 | 0.675 |
| 20% | 0.719 | 0.676 | 0.750 | 0.683 |
| 25% | 0.726 | 0.682 | 0.782 | 0.689 |
| 30% | 0.705 | 0.708 | * | 0.716 |
| *Too few positive for emphysema. All area under the curves were significantly different from 0.50 (p-value range <0.0001 - <0.05). | | | | |

| **Table S4** ROC area under the curves for visual emphysema (present/absent) and the different sRAGE platforms | | | |
| --- | --- | --- | --- |
| Cohort | N | Biomarker | Area Under the Curve |
| COPDGene | 499 | LCMS | 0.572* |
| COPDGene | 1,376 | QBR | 0.531 |
| COPDGene | 580 | RBM | 0.627 |
| COPDGene | 1,177 | SOMAscan | 0.560 |
| ECLIPSE | 1,971 | QBR | 0.541* |
| SCCOR | 399 | DuoSet | 0.521* |
| SPIROMICS | 869 | RBM | 0.578 |
| *Area under the curves are not significantly different from 0.50, rest (p-value range 0.02 - <0.0001) | | | |

| **Table S5.** ROC for the different emphysema cut-points and DLco percent predicted | | | | | |
| --- | --- | --- | --- | --- | --- |
|  | | Area Under the Curves | | |  |
|  |  | COPDGene at Visit 2 | | |  |
| % Emphysema cut-points | Subset of QBR visit 1 observations  n=767 | All observation at Visit 2 n=4,624 |  |  |  |
| 5% | 0.82 | 0.78 |  |  |  |
| 10% | 0.89 | 0.86 |  |  |  |
| 15% | 0.94 | 0.90 |  |  |  |
| 20% | 0.95 | 0.93 |  |  |  |
| 25% | 0.97 | 0.95 |  |  |  |
| 30% | 0.97 | 0.96 |  |  |  |
| All area under the curves were significantly different from 0.50 (p<0.0001) | | |  |  |  |

**References**

1. Hoffman EA, Ahmed FS, Baumhauer H, Budoff M, Carr JJ, Kronmal R, Reddy S, Barr RG. Variation in the percent of emphysema-like lung in a healthy, nonsmoking multiethnic sample. The MESA lung study. *Ann Am Thorac Soc* 2014; 11: 898-907.

2. Lynch DA, Moore CM, Wilson C, Nevrekar D, Jennermann T, Humphries SM, Austin JHM, Grenier PA, Kauczor HU, Han MK, Regan EA, Make BJ, Bowler RP, Beaty TH, Curran-Everett D, Hokanson JE, Curtis JL, Silverman EK, Crapo JD, Genetic Epidemiology of CI. CT-based Visual Classification of Emphysema: Association with Mortality in the COPDGene Study. *Radiology* 2018; 288: 859-866.

3. Gietema HA, Muller NL, Fauerbach PV, Sharma S, Edwards LD, Camp PG, Coxson HO, Evaluation of CLtIPSEi. Quantifying the extent of emphysema: factors associated with radiologists' estimations and quantitative indices of emphysema severity using the ECLIPSE cohort. *Acad Radiol* 2011; 18: 661-671.

4. Ajala O, Zhang Y, Gupta A, Bon J, Sciurba F, Chandra D. Decreased serum TRAIL is associated with increased mortality in smokers with comorbid emphysema and coronary artery disease. *Respiratory medicine* 2018; 145: 21-27.

5. Cho MH, McDonald ML, Zhou X, Mattheisen M, Castaldi PJ, Hersh CP, Demeo DL, Sylvia JS, Ziniti J, Laird NM, Lange C, Litonjua AA, Sparrow D, Casaburi R, Barr RG, Regan EA, Make BJ, Hokanson JE, Lutz S, Dudenkov TM, Farzadegan H, Hetmanski JB, Tal-Singer R, Lomas DA, Bakke P, Gulsvik A, Crapo JD, Silverman EK, Beaty TH, Nett Genetics IE, Investigators CO. Risk loci for chronic obstructive pulmonary disease: a genome-wide association study and meta-analysis. *The Lancet Respiratory medicine* 2014; 2: 214-225.

6. Sun W, Kechris K, Jacobson S, Drummond MB, Hawkins GA, Yang J, Chen TH, Quibrera PM, Anderson W, Barr RG, Basta PV, Bleecker ER, Beaty T, Casaburi R, Castaldi P, Cho MH, Comellas A, Crapo JD, Criner G, Demeo D, Christenson SA, Couper DJ, Curtis JL, Doerschuk CM, Freeman CM, Gouskova NA, Han MK, Hanania NA, Hansel NN, Hersh CP, Hoffman EA, Kaner RJ, Kanner RE, Kleerup EC, Lutz S, Martinez FJ, Meyers DA, Peters SP, Regan EA, Rennard SI, Scholand MB, Silverman EK, Woodruff PG, O'Neal WK, Bowler RP, Group SR, Investigators CO. Common Genetic Polymorphisms Influence Blood Biomarker Measurements in COPD. *PLoS Genet* 2016; 12: e1006011.

7. Pillai SG, Kong X, Edwards LD, Cho MH, Anderson WH, Coxson HO, Lomas DA, Silverman EK, Eclipse, Investigators I. Loci identified by genome-wide association studies influence different disease-related phenotypes in chronic obstructive pulmonary disease. *Am J Respir Crit Care Med* 2010; 182: 1498-1505.

8. Coxson HO, Dirksen A, Edwards LD, Yates JC, Agusti A, Bakke P, Calverley PM, Celli B, Crim C, Duvoix A, Fauerbach PN, Lomas DA, Macnee W, Mayer RJ, Miller BE, Muller NL, Rennard SI, Silverman EK, Tal-Singer R, Wouters EF, Vestbo J, Evaluation of CLtIPSEI. The presence and progression of emphysema in COPD as determined by CT scanning and biomarker expression: a prospective analysis from the ECLIPSE study. *The Lancet Respiratory medicine* 2013; 1: 129-136.

| **Section/topic** | **#** | **Checklist item** | **Reported on page #** |
| --- | --- | --- | --- |
| **TITLE** | | |  |
| Title | 1 | Identify the report as a systematic review, meta-analysis, or both. | NA |
| **ABSTRACT** | | |  |
| Structured summary | 2 | Provide a structured summary including, as applicable: background; objectives; data sources; study eligibility criteria, participants, and interventions; study appraisal and synthesis methods; results; limitations; conclusions and implications of key findings; systematic review registration number. | NA |
| **INTRODUCTION** | | |  |
| Rationale | 3 | Describe the rationale for the review in the context of what is already known. | 4-6 |
| Objectives | 4 | Provide an explicit statement of questions being addressed with reference to participants, interventions, comparisons, outcomes, and study design (PICOS). | 6 |
| **METHODS** | | |  |
| Protocol and registration | 5 | Indicate if a review protocol exists, if and where it can be accessed (e.g., Web address), and, if available, provide registration information including registration number. | NA |
| Eligibility criteria | 6 | Specify study characteristics (e.g., PICOS, length of follow-up) and report characteristics (e.g., years considered, language, publication status) used as criteria for eligibility, giving rationale. | NA |
| Information sources | 7 | Describe all information sources (e.g., databases with dates of coverage, contact with study authors to identify additional studies) in the search and date last searched. | NA |
| Search | 8 | Present full electronic search strategy for at least one database, including any limits used, such that it could be repeated. | NA |
| Study selection | 9 | State the process for selecting studies (i.e., screening, eligibility, included in systematic review, and, if applicable, included in the meta-analysis). | NA |
| Data collection process | 10 | Describe method of data extraction from reports (e.g., piloted forms, independently, in duplicate) and any processes for obtaining and confirming data from investigators. | NA |
| Data items | 11 | List and define all variables for which data were sought (e.g., PICOS, funding sources) and any assumptions and simplifications made. | NA |
| Risk of bias in individual studies | 12 | Describe methods used for assessing risk of bias of individual studies (including specification of whether this was done at the study or outcome level), and how this information is to be used in any data synthesis. | NA |
| Summary measures | 13 | State the principal summary measures (e.g., risk ratio, difference in means). | 11-12 |
| Synthesis of results | 14 | Describe the methods of handling data and combining results of studies, if done, including measures of consistency (e.g., I^2^) for each meta-analysis. | Supplement  3-4 |

Page 1 of 2

| **Section/topic** | **#** | **Checklist item** | **Reported on page #** |
| --- | --- | --- | --- |
| Risk of bias across studies | 15 | Specify any assessment of risk of bias that may affect the cumulative evidence (e.g., publication bias, selective reporting within studies). | NA |
| Additional analyses | 16 | Describe methods of additional analyses (e.g., sensitivity or subgroup analyses, meta-regression), if done, indicating which were pre-specified. | NA |
| **RESULTS** | | |  |
| Study selection | 17 | Give numbers of studies screened, assessed for eligibility, and included in the review, with reasons for exclusions at each stage, ideally with a flow diagram. | NA |
| Study characteristics | 18 | For each study, present characteristics for which data were extracted (e.g., study size, PICOS, follow-up period) and provide the citations. | 6-7 |
| Risk of bias within studies | 19 | Present data on risk of bias of each study and, if available, any outcome level assessment (see item 12). | NA |
| Results of individual studies | 20 | For all outcomes considered (benefits or harms), present, for each study: (a) simple summary data for each intervention group (b) effect estimates and confidence intervals, ideally with a forest plot. | 11-12 |
| Synthesis of results | 21 | Present results of each meta-analysis done, including confidence intervals and measures of consistency. | 11-12 |
| Risk of bias across studies | 22 | Present results of any assessment of risk of bias across studies (see Item 15). | NA |
| Additional analysis | 23 | Give results of additional analyses, if done (e.g., sensitivity or subgroup analyses, meta-regression [see Item 16]). | NA |
| **DISCUSSION** | | |  |
| Summary of evidence | 24 | Summarize the main findings including the strength of evidence for each main outcome; consider their relevance to key groups (e.g., healthcare providers, users, and policy makers). | NA |
| Limitations | 25 | Discuss limitations at study and outcome level (e.g., risk of bias), and at review-level (e.g., incomplete retrieval of identified research, reporting bias). | 15 |
| Conclusions | 26 | Provide a general interpretation of the results in the context of other evidence, and implications for future research. | 17 |
| **FUNDING** | | |  |
| Funding | 27 | Describe sources of funding for the systematic review and other support (e.g., supply of data); role of funders for the systematic review. | NA |

*From:*  Moher D, Liberati A, Tetzlaff J, Altman DG, The PRISMA Group (2009). Preferred Reporting Items for Systematic Reviews and Meta-Analyses: The PRISMA Statement. PLoS Med 6(7): e1000097. doi:10.1371/journal.pmed1000097

For more information, visit: **www.prisma-statement.org**.

Page 2 of 2
